# Fully Breaking Entanglement of Multiple Harmonics for Space‐ and Frequency‐Division Multiplexing Wireless Applications via Space‐Time‐Coding Metasurface

**DOI:** 10.1002/advs.202404558

**Published:** 2024-07-04

**Authors:** Zhangjie Luo, Zhiming Zhang, Junwei Tai, Lei Zhang, Chenglong Gao, Hui Feng Ma, Wei Xiang Jiang, Qiang Cheng, Tie Jun Cui

**Affiliations:** ^1^ State Key Laboratory of Millimeter Waves School of Information Science and Engineering Southeast University Nanjing 210096 China; ^2^ Institute of Electromagnetic Space Southeast University Nanjing 210096 China

**Keywords:** disentanglement, nonlinear harmonic generation, space‐ and frequency‐division multiplexing, space‐time‐coding metasurface, wireless communication

## Abstract

Harmonic generation and utilization are significant topics in nonlinear science. Although the progress in the microwave region has been expedited by the development of time‐modulated metasurfaces, one major issue of these devices is the strong entanglement of multiple harmonics, leading to criticism of their use in frequency‐division multiplexing (FDM) applications. Previous studies have attempted to overcome this limitation, but they suffer from designing complexity or insufficient controlling capability. Here a new space‐time‐coding metasurface (STCM) is proposed to independently and precisely synthesize not only the phases but also the amplitudes of various harmonics. This promising feature is successfully demonstrated in wireless space‐ and frequency‐division multiplexing experiments, where modulated and unmodulated signals are simultaneously transmitted via different harmonics using a shared STCM. To illustrate the advantages, binary frequency shift keying (BFSK) and quadrature phase shift keying (QPSK) modulation schemes are respectively implemented. Behind the intriguing functionality, the mechanism of the space‐time coding strategy and the analytical designing method are elaborated, which are validated numerically and experimentally. It is believed that the achievements can potentially propel the time‐vary metasurfaces in the next‐generation wireless applications.

## Graphical abstract

This work presents a novel space‐time‐coding metasurface to disentangle the amplitudes and phases of multiple harmonics, providing an effective approach to address the long‐standing challenge of time‐varying metasurfaces. The proposed temporal coding strategy is analytically derived, which makes the designing process simple and efficient. It allows for the maximum exploitation of spatial modulation for space‐ and frequency‐division multiplexing applications.

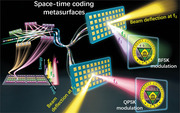

## 1 Introduction

Nonlinear science is a prominent subject in contemporary physics, which has led to a diverse range of advanced technologies and applications.^[^ ^1^, ^2^, ^3^, ^4^, ^5^, ^6^ ^]^ However, while nonlinear optics has been advancing for decades, the study of nonlinear effects in the microwave region has remained stagnant until the emergence of metamaterials.^[^ ^7^, ^8^, ^9^, ^10^, ^11^, ^12^, ^13^, ^14^, ^15^, ^16^, ^17^, ^18^ ^]^ Over the past 20 years, the investigations of metamaterials have experienced a significant surge in development, resulting in the discovery of many new and intriguing nonlinear phenomena with potential applications, such as harmonic generation,^[^ ^19^, ^20^, ^21^ ^]^ wave mixing,^[^ ^22^ ^]^ resonance shifting,^[^ ^23^, ^24^, ^25^ ^]^ and energy‐selective reflection/transmission/absorption,^[^ ^26^, ^27^, ^28^, ^29^, ^30^, ^31^, ^32^ ^]^ to name a few.

Conventional methods for generating harmonics typically depend on the interactions between the concentrated electromagnetic (EM) fields and nonlinear components embedded in the metamaterials.^[^ ^19^, ^20^ ^]^ Recent advancements have shown that the incoming fundamental‐frequency (FF) waves can be converted into a sequence of sideband signals by altering the EM properties of a metasurface over time.^[^ ^21^, ^33^, ^34^, ^35^, ^36^, ^37^, ^38^, ^39^, ^40^, ^41^, ^42^, ^43^, ^44^, ^45^, ^46^, ^47^, ^48^, ^49^, ^50^ ^]^ When further combined with spatial modulations, the space‐time modulated metasurfaces offer a family of fascinating harmonic applications involving nonreciprocity,^[^ ^37^, ^38^, ^39^, ^40^, ^41^, ^42^, ^43^ ^]^ waveform modulation,^[^ ^44^, ^45^, ^46^ ^]^ polarization regulation,^[^ ^47^, ^48^, ^49^ ^]^ spectral camouflage,^[^ ^50^ ^]^ and so on. Thus, a broad avenue for the exploitation of these nonlinear behaviors has been opened up.

Digital programmable metasurfaces offer a practical solution to realize the time‐varying modulation for harmonic generations.^[^ ^21^, ^42^, ^45^, ^46^, ^47^, ^48^, ^49^ ^]^ Composed of a lattice of reconfigurable unit cells that can be controlled by digital modules,^[^ ^12^, ^51^ ^]^ the programmable metasurface has attracted growing interest owing to the ability to dynamically tailor EM wave properties, which leads to various advanced functionalities.^[^ ^52^, ^53^, ^54^, ^55^, ^56^, ^57^, ^58^, ^59^ ^]^ Since the coding sequences provided by the digital modules are not only the controlling commands but also information streams that can be modulated on the EM waves, the programmable metasurface innovatively bridges the EM world and the information world, hence opening up possibilities for novel wireless communication systems that are much less complex than traditional architectures.^[^ ^60^, ^61^ ^]^ Based on this idea, the harmonics generated by the time‐modulated programmable metasurface are further employed as carrier waves for wireless communications. High‐order modulation schemes and space‐division multiplexing (SDM) were successively reported,^[^ ^62^, ^63^, ^64^ ^]^ suggesting an innovative way for the next‐generation wireless communication systems.

Owing to the capability to generate multiple harmonics, the time‐modulated programmable metasurface has shown great potential for frequency‐division multiplexed (FDM) applications. However, it is criticized due to the inherent entanglement of these harmonics. Specifically, it is difficult to independently control the amplitudes and phases of certain harmonics without interference with others. In the early stage, optimization algorithms were employed to conquer this limitation, yet at the expense of significant computational cost.^[^ ^45^ ^]^ Later, a recipe was offered by engineering the modulation sequences with switchable phase states and specific time delays, but the number of harmonics that can be independently controlled with disentangled amplitude and phase were one^[^ ^65^ ^]^ and two.^[^ ^66^ ^]^ Recently, an asynchronous approach was proposed for multi‐harmonic manipulations.^[^ ^67^ ^]^ The metasurface was divided into several partitions, and the unit cells within each partition were modulated by a particular frequency. Therefore, the frequencies of the harmonics were independently designed, and their amplitudes could be realized by changing the area ratio of the partitions. Decreasing the amplitude of a certain harmonic would reduce the ability to manipulate its scattering property. This was proved by the work in ref. [68] where only a few cells were employed to generate the +1st order harmonic; as a result, the energy was scattered without any specific directionality. Another coding solution to disentangle the phases of multiple harmonics was proposed by temporally intertwining a series of sub‐sequences (SSs).^[^ ^69^ ^]^ However, more complex coding methods were required for the amplitude synthesis, which was unfortunately not provided in the literature. Hence, to fully disentangle the multiple harmonics with a simple and cost‐effective solution remains a challenge. This arouses the motivation for this work.

In this paper, we propose a space‐time‐coding metasurface (STCM) to disentangle the multiple harmonics and demonstrate its advanced utilization in space‐ and frequency‐division multiplexing (SFDM) wireless applications. The characteristics of this work are described in the following aspects. Most importantly, we focus on the independent synthesis of not only phases but also amplitudes of multiple harmonics, which can significantly extend the ability for advanced harmonic applications. Secondly, this approach relies only on the proposed temporal coding strategy, so it allows for the maximum exploitation of spatial modulation for SFDM. Thirdly, the design process of the coding sequences is based on analytical derivations, making it simple and avoiding the need for complex optimizations. Furthermore, the controlling module includes only a field programmable gate array (FPGA) and driving circuit, which is cost‐effective compared to those with expensive digital‐analog converters and amplifiers. In Note S1 (Supporting Information), the proposed STCM and those in previous studies are compared in detail, and the advantages of this work can be found in terms of cost‐effectiveness and simpleness.

**Figure 1:**
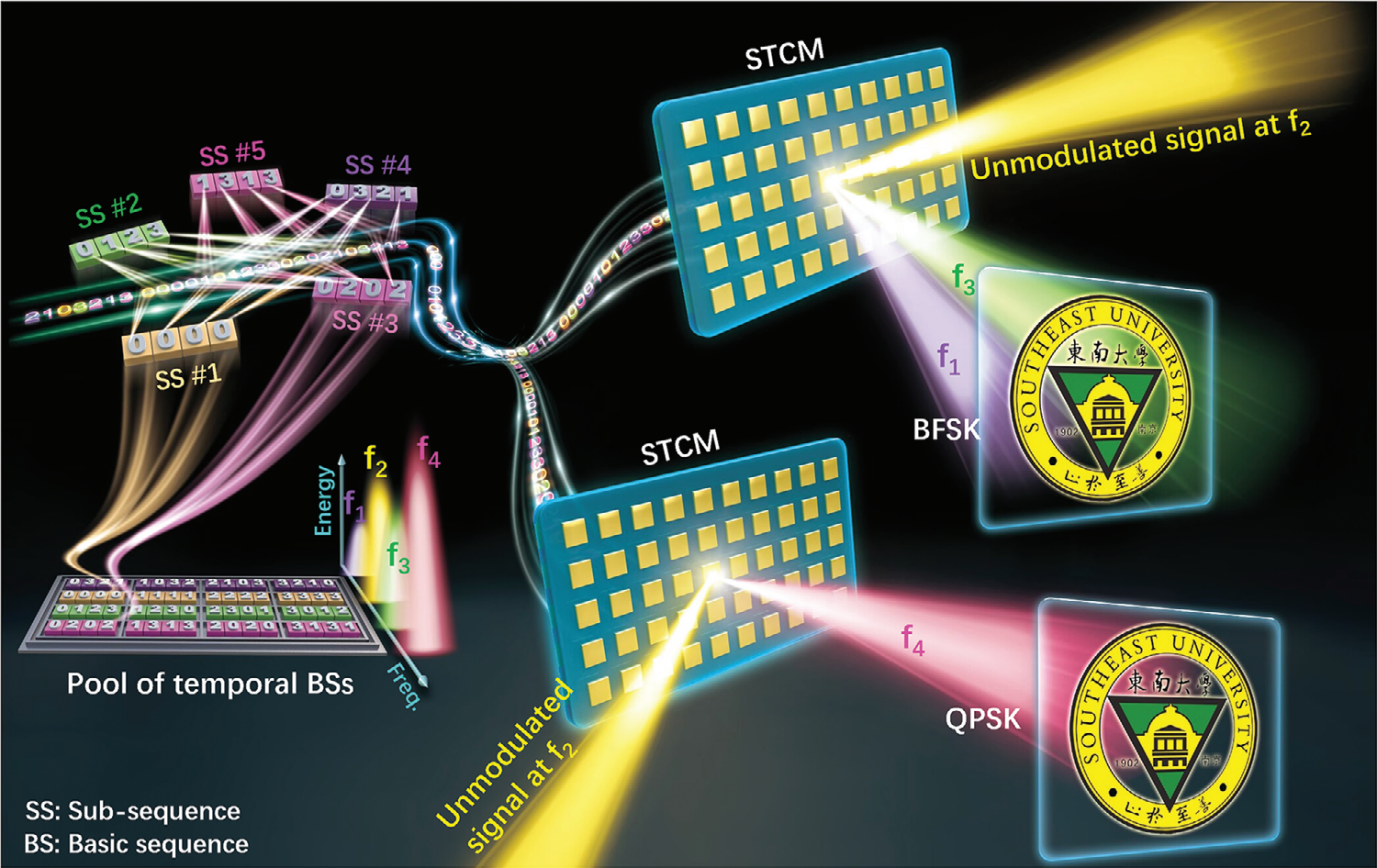
Concept of the proposed STCM. The STCM is capable of fully breaking the entanglement of the amplitudes and phases at different harmonics. The coding sequence is composed of interweaved sub‐sequences (SSs). Distinguished from previous works, the SSs are selected from a pool of exhaustively listed basic sequences (BSs), which play a key role in the harmonic generations and their manipulations. In this illustration using the 2‐bit STCM, up to four harmonics can be controlled simultaneously and independently. To validate the concept, precise harmonic beam‐steering phenomena are realized using STCM, and a hybrid wireless transmitter is built for simultaneous wireless modulated and unmodulated signal transmissions at various harmonic frequencies. Two modulation schemes, BFSK and QPSK, are realized.

Based on the theoretical achievements, we employ the STCM as a wireless transmitter that can simultaneously transmit modulated and unmodulated signals via separated frequencies and in different directions, as illustrated in **Figure** **1**. Two modulation schemes, i.e., binary frequency‐shift keying (BFSK) and quadrature phase shift keying (QPSK), are respectively implemented to showcase the advantages of our method. Figure 1 also illustrates how the proposed temporal coding strategy is implemented, showing that the coding sequence is constructed by interweaving a series of SSs. Although it may appear similar to the method described in the literature,^[^ ^69^ ^]^ it differs not only in its theoretical analysis but also in the process of designing the sequence. As shown in the figure, the SSs are selected from a pool of pre‐designed basic sequences (BSs). Each BS in the time domain is responsible for generating a unique harmonic component in the frequency domain. All the harmonics offered by the BSs are orthogonal in the frequency domain. By purposely picking the BSs, determining their indices as the SSs, and intertwining them to build a controlling sequence, target harmonics can be generated with the desired amplitudes and phases without interferences. These are all accomplished in the time domain. Further combined with the spatial modulation, the precise controllability of the STCM over the harmonic beam magnitudes and orientations can be realized. Because the BSs are pre‐designed through the analytical method and exhaustively listed in the pool, the design process of the coding sequences is greatly simplified. In the following paragraphs of this paper, the theory and designing method will be introduced in detail; to verify the theory, the disentangled manipulations of multiple harmonic beams will be discussed sufficiently through simulations and measurements; finally, the hybrid wireless experiments and discussions will be presented.

## 2 Theory and Simulations

### 2.1 Theory of the Temporal Coding Strategy

We consider a reflective STCM illuminated normally by an EM wave in the time domain. The reflection coefficient of the metasurface Γ(*t*) is a periodic function of time. Here, we consider the phase modulation, which means that the reflection magnitudes are assumed to be uniform, and the reflection phases, which are described by binary codings, vary with time. According to the previous works of literature, by purposely designing the coding strategy, the sideband signals can be generated and manipulated.^[^ ^45^ ^]^ In the following paragraphs, we introduce the theory of the proposed modulation strategy.

**Figure 2:**
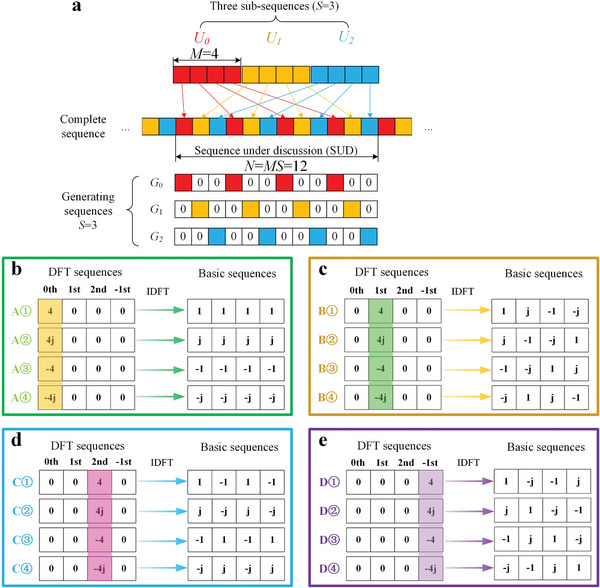
Theories of the coding strategy. a) Schematic illustration of the intertwined coding strategy. In this figure, three SSs (*U*_0_, *U*_1_, *U*_2_) with a length of four are employed to comprise the sequence under discussion (SUD) by interweaving their coding states successively. The complete coding sequence is created by periodically repeating the SUD. The generating sequences (GSs) are introduced to help the derivation, and the SUD can be presented by summing all the GSs. b–e) The pool of the BSs to generate the four harmonics using the 2‐bit STCM. These BSs are obtained through inverse discrete Fourier transform (IDFT) of the DFT sequences. The DFT sequences corresponding to a specific harmonic are exhaustively listed, which are required to be orthogonal in the frequency domain. b) 0th harmonic. c) +1st harmonic. d) +2nd harmonic. e) −1st harmonic.

Without loss of generality, we consider a *q*‐bit reflective coding metasurface, and the number of reflection phases, called the coding states, is 2^*q*^. The complete coding sequence is built up by periodically repeating the sequence under discussion (SUD). The SUD is created by intertwining a series of *S* SSs of length *M*. As proved in Note S2 (Supporting Information), *M* is limited by 2^*q*^. Here, we let *M* equal 2^*q*^, so the length of SUD *N* = *MS*. The *n*‐th coding state of the SUD is denoted as Γ(*n*), 0 ≤ *n* ≤ *N* − 1. The *i*‐th SS is denoted as *U_i_*,0 ≤ *i* ≤ *S* − 1. The intertwining principle is the same as the one presented in ref. [69] which is briefly illustrated in **Figure** **2**a. In this figure, we use three SSs (*S* = 3) of length *M* = 4 to compose the SUD, by successively inserting their coding states into the correct positions in an intertwining way. The length of the SUD *N* = *MS* = 12. It is worth noting that the indices and colors of the SSs in the figure are used to explain the intertwining principle, which is unrelated to their actual codings. In other words, SSs with different index numbers or colors can be the same or different, which are determined by the harmonic manipulation goals.

Beyond the literature,^[^ ^69^ ^]^ we innovatively introduce the generating sequences (GSs) of the SSs to help the formula derivation. Each GS corresponds to an SS, so their number is also *S*. The length of each GS is *N*, the same as that of the SUD. The *n*‐th coding state of the *i*‐th GS is denoted as *G_i_* (*n*), 0 ≤ *n* ≤ *N* − 1, 0 ≤ *i* ≤ *S* − 1, which is described by

(1) $$G_{i}\left(n\right)\left\{\begin{array}{c} U_{i}\left(m\right),\;\mathrm{when}\;n=S\cdot m+i\\ 0,\;\;\;\;\mathrm{otherwise} \end{array}\right.,\;0\leq m\leq M-1$$

Therefore, the *n*‐th coding state of the SUD can be presented by the sum of the *n*‐th coding states of all the GSs, as shown in Figure 2a, that is,

(2) $$\Gamma \left(n\right)=\sum_{i=0}^{S-1}G_{i}\left(n\right),\;0\leq n\leq N-1$$

This is the significance of the introduction of the GSs. Then, we derive the discrete Fourier transform (DFT) of the GS, *G_i_* (*n*), in Equation (1), which is

(3) $$\begin{array}{c} F_{i}\left(k\right)=\sum_{n=0}^{N-1}G_{i}\left(n\right)\exp\left(-j\frac{2kn\pi }{N}\right)\\ =P_{i}\left(r\right)\exp\left(-j2\pi \frac{ik}{N}\right)\\ =P_{i}\left(k\;\mathrm{mod}\;\;\;M\right)\exp\left(-j2\pi \frac{ik}{N}\right) \end{array}$$

Here *k* = *Ms* + *r*, *s* = 0, 1,…, *S* − 1; *r* = 0, 1,…, *M* − 1. *P_i_* (*r*) = *FFT*[*U_i_* (*m*)] is the DFT of the *i*‐th SS. The derivation of Equation (3) is presented in Note S3 (Supporting Information).

According to Equation (3), the relation between the *k*‐th order harmonic and the *i*‐th SS is established. By performing the DFT operations to Equation (2) and plugging the result into Equation (3), we have the harmonics generated by the SUD, that is,

(4) $$\begin{array}{c} TF\left(k\right)=FFT\left[\Gamma_{n}\right]=\sum_{i=0}^{S-1}F_{i}\left(k\right)=\sum_{i=0}^{S-1}\left\{P_{i}\left(k\;\mathrm{mod}\;\;\;M\right)\exp\left(-j2\pi \frac{ik}{N}\right)\right\} \end{array}$$

Notice that (kmodM)=r, indicating that the maximum number of controllable harmonics is 2^*q*^, while the syntheses of other harmonics repeat periodically with a period of 2^*q*^ and with progressively decreasing strengths.

From Equation (4), it is also concluded that the DFT result of the SUD is the vector superposition of the DFT results of all SSs that comprise the SUD. As a result, not only the phases but also the amplitudes of various harmonics can be flexibly and independently controlled by purposely designing the SSs to build the SUD. This provides a deep understanding of the harmonic regulations, laying the theoretical foundation of our work.

### 2.2 Basic Sequences

In this study, SSs for the harmonic controls are chosen from a pool of temporal BSs, which are designed in advance, as shown in Figure 1. These BSs are produced by starting with their DFT sequences in the frequency domain. Considering an STCM with 2^*q*^ coding states, the number of DFT sequences is *M*, and their length is *M* (*M* = 2^*q*^). Each DFT sequence corresponds to a specific harmonic order, so the number of controllable harmonics is 2^*q*^, and they should be orthogonal in the frequency domain. The DFT sequences are exhaustively listed as $X(0)=\left[Me^{j\varphi },0,0,\text{…},0\right]$, $X(1)=\left[0,Me^{j\varphi },0,\text{…},0\right]$, …, $X(M-2)=\left[0,\text{…},0,Me^{j\varphi },0\right]$, $X(M-1)=\left[0,\text{…},0,Me^{j\varphi }\right]$. For each DFT sequence, there are *M* = 2^*q*^ values for the phase *φ*, which are $\varphi_{0}=\frac{2\pi \cdot 0}{M}$, $\varphi_{1}=\frac{2\pi \cdot 1}{M}$,…, $\varphi_{M-2}=\frac{2\pi \cdot (M-2)}{M}$, $\varphi_{M-1}=\frac{2\pi \cdot (M-1)}{M}$. Constrained by the orthogonality in the frequency domain, the number of all BSs is *M* ^2^.

Then, the temporal BSs for each harmonic are obtained through inverse DFT (IDFT) operations. This approach is particularly efficient in design when dealing with programmable metasurfaces that have a finite number of coding states. It allows for the exhaustive listing of all BSs and their DFT sequences in a pool for harmonic syntheses. Figure 2b–e presents all the DFT sequences and corresponding BSs of a 2‐bit metasurface. In Figure 2b, four BSs responsible for generating four 0th harmonic components are provided. The four harmonic components have the same amplitude of 4 and different phases with an interval of 90°. In the same manner, the BSs and corresponding DFT sequences for the +1st, +2nd, and −1st harmonics are listed in Figure 2c–e. In the following paragraphs, BSs are selected from this pool as SSs to build the SUD to manipulate amplitudes and phases of target harmonics, as shown in Figure 1. It should be mentioned that the frequency of the 0th harmonic is the same as the FF, but it is generated by the nonlinear interactions between the incident FF wave and the STCM.

### 2.3 Independent Syntheses of Multiple Harmonics’ Amplitudes

According to Equation (4), the *k*‐th order harmonic generated by the SUD is the vector superposition of the *k*‐th order harmonic components generated by the SSs that build the SUD. In other words, the amplitude ratio of harmonics is determined by the number of their BSs chosen as the SSs as well as their indices. With a 2‐bit STCM, amplitudes of up to four harmonics can be simultaneously and independently controlled. To elucidate the mechanism, we take the 0th and +1st harmonics for example. The BS A①[1, 1, 1, 1] in Figure 2b is chosen for the 0th harmonic, and B①[1, *j*, −1, ‐*j*] in Figure 2c is chosen for the +1st harmonic. Their DFT sequences are [4,0,0,0] and [0,4,0,0], respectively, which means that the amplitudes of the harmonics are 4, and their phases, called initial phases φ_initial_, are 0°.

**Figure 3:**
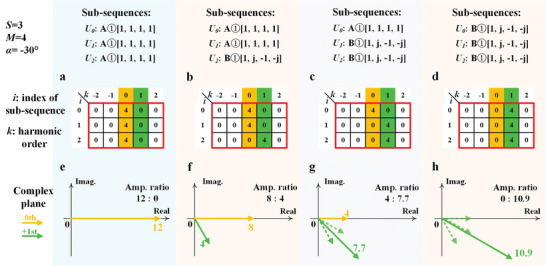
Independent amplitude syntheses of the 0th and +1st harmonics. The BS A①[1, 1, 1, 1] for the 0th harmonic and BS B①[1, *j*, −1, ‐*j*] for the +1th harmonic are selected as the SSs to compose the SUD with four different combinations. a) All the SSs are A①. b) The first two SSs are A①, and the last one is B①. c) The first SS is A①, and the last two are B①. d) All the SSs are B①. e–h) The harmonic vectors in the complex plane, which are the superpositions of the harmonic vectors generated by the SSs.

Three SSs (*S* = 3) are adopted for the amplitude synthesis. **Figure** **3** gives the four combinations of the two selected BSs that comprise the SUD. In the *i* − *k* tables, *i* = 0, 1, 2 is the index of the SS, and *k* = −2, −1, 0, 1, 2 is the harmonic order. Each row of the red frame shows that the *i*‐th SS contributes to the amplitude of the corresponding harmonic. The phase of the *k*‐th order harmonic offered by the *i*‐th SS

(5) $$\varphi_{i}=\varphi_{\mathrm{initial}\#i}+ik\alpha$$

Here the term α = −2π/*N* is the term − 2π/*N*in Equation (4), and *N* is the length of the SUD. In this example, *N* = 12, so α = −30°, and $\varphi_{\mathrm{initial}\#i}=0^{\circ }$. In the complex planes in Figure 3e–h, the generated 0th (orange vector) or +1st (green vector) harmonics are plotted, which are the superpositions of the harmonic vectors contributed by the SSs. In Figure 3a, the three SSs are the same BS A①[1, 1, 1, 1]. The corresponding *i* − *k* table shows that only the 0th harmonic is produced, and the amplitude produced by each SS is 4, so the synthetic amplitude is 12. For each SS, φ_*i*_ = 0°, so the phase of the 0th harmonic is 0°, as shown in Figure 3e. In Figure 3b, the SSs *U_0_* and *U_1_* are BS A①[1, 1, 1, 1], and the SS *U_2_* is BS B①[1, *j*, −1, ‐*j*]. For each SS, the phases of the first two SSsφ_0_ = φ_1_ = 0°, and the phase of the third SS $\varphi_{2}=\varphi_{\mathrm{initial}\#2}+2\times 1\times \alpha =0^{\circ }+\left(-60^{\circ }\right)=-60^{\circ }$. As a result, the phases of the 0th and +1st harmonics are 0° and −60°, respectively, and the amplitude ratio of the 0th and +1st harmonics is 8:4, as plotted in Figure 3f. In Figure 3c, the SS *U*_0_ is A①[1, 1, 1, 1], and the SSs *U*_1_ and *U*_2_ are B①[1, *j*, −1, ‐*j*]. Following the above analysis, we can have the amplitude ratio of the 0th and +1st harmonics to be 4:7.7, as shown in Figure 3g. With all the SSs set as B①[1, *j*, −1, ‐*j*] in the last case in Figure 3d, the 0th harmonic is not generated. The +1st harmonic vector is the superposition of the three vectors, which have the same amplitude and three phases of −0°, −30°, and −60°, respectively. As a result, the phase of the harmonic vector is −30°, and the amplitude is 10.9, as shown in Figure 3h.

In the above examples, three SSs (*S* = 3) are utilized to control the two harmonics, so four combinations of the SSs are presented for four amplitude ratios. There are two ways to adjust the ratio of target harmonics with a higher precision. The first one is straightforward in the time domain by increasing the number of SSs and picking up more specific BSs as SSs to form a longer coding sequence. However, this will result in a smaller frequency interval between the harmonics if the switching frequency of the controlling signal is fixed. The second approach is realized by introducing a space‐time modulation on the metasurface. This will be illustrated in Section 3.1 (3) and Note S6 (Supporting Information).

### 2.4 Independent Syntheses of Multiple Harmonic Phases

**Figure 4:**
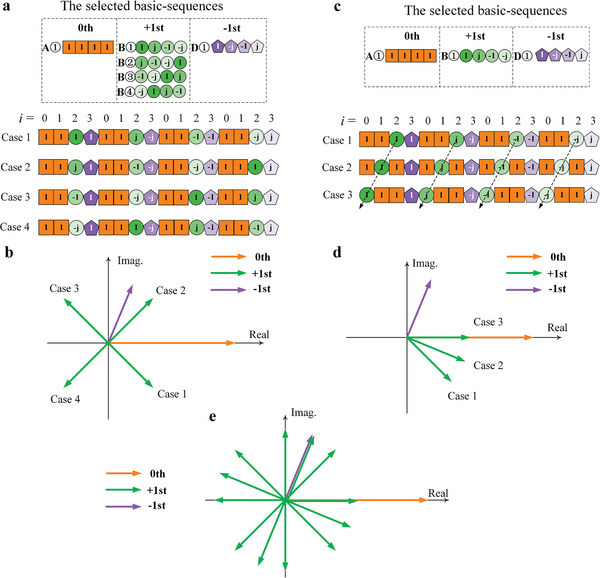
Independent phase synthesis of the +1st harmonic. During the process, the phases of the 0th and −1st harmonics and the amplitudes of the three harmonics remain fixed. a) The mechanism of the phase control by selecting different BSs of the +1st harmonic, which generate the harmonic with different initial phases. b) Four vectors of the +1st harmonic generated in the four cases, showing the phases of −45°, 45°, 135°, and 225°, respectively. c) The mechanism of the phase control by switching the index of the BSs of the +1st and 0th harmonics. d) Three vectors of the +1st harmonic generated in the three cases, showing the phases of −45°, −22.5°, and 0°, respectively. e) Twelve vectors of the +1st harmonic, which are obtained by combining the above two methods. More details about the implementation are presented in Note S4 (Supporting Information).

In the above section, we discussed the method to synthesize the amplitude ratio of the multiple harmonics. It should be noted that their phases may also undergo changes as their amplitudes are adjusted, as shown by the green vectors in Figure 3f–h. In this section, we will explain the method to synthesize their phases once their amplitude ratio has been fixed. According to Equation (5), $\varphi_{i}=\varphi_{\mathrm{initial}\#i}+ik\alpha$, two factors are provided to control the target harmonic phase. The first one is the initial phase φ_initial_ of each harmonic component, and the other is the index *i* of each SS in the SUD. **Figure** **4** illustrates how the phase of the +1st harmonic can be manipulated independently without altering the phases of the −1st and 0th harmonics or the amplitudes of any of the three harmonics. The number of SSs is four (*S* = 4), and the SS length is four (*M* = 4), so the length of the SUD is sixteen (*N* = 16). Two BSs A①[1, 1, 1, 1] are included to generate the 0th harmonic, and one BS D①[1, ‐*j*, −1, *j*] is selected to generate the −1st harmonic.

1. Synthesize the phase of the +1st harmonic by selecting its BSs.

The key here is to change the initial phase φ_initial_ of the harmonic component. Figure 4a,b displays the mechanism. BSs B①, B②, B③, and B④ are ready to be chosen in the four cases, whose initial phases are 0°, 90°, 180°, and 270°, respectively. Because the numbers of BSs are determined, the amplitude ratio of the three harmonics is fixed. The SS indices for the four SSs are fixed, so the phase of the −1st harmonic is constant, that is, φ_‐1st_ = 0° + 3 × (− 1) × (− 22.5°) = 67.5°. Since the order *k* is 0 for the 0th harmonic, its phase keeps 0°, as shown in Figure 4b. In Case 1, the initial phase of the +1st harmonic generated by B① is 0°, so the phase φ_+ 1st_ = 0° + 2 × (+ 1) × (− 22.5°) = −45°; in Case 2, the initial phase of the +1st harmonic generated by B② is 90°, so the phase φ_+ 1st_ = 90° + 2 × (+ 1) × (− 22.5°) = 45°. In the same manner, the phases in Cases 3 and 4 are 135° and 225°, respectively. Therefore, by changing the BSs of the +1st harmonic, its phase can be altered with an interval of 90°, as shown by the green vectors in Figure 4b.

1. Synthesize the phase of the +1st harmonic by switching the index of its BS, *i*, with that of the 0th harmonic in the SUD.

As shown in Figure 4c, the BS is fixed to be B① for example. During this process, because the order of the 0th harmonic *k* is 0, its phase does not change. Meanwhile, the index of the BS of the −1st harmonic remains the same, so its phase does not change. Their vectors are fixed, as plotted in Figure 4d. The phases of the +1st harmonic in the three cases in Figure 4c can be easily calculated using Equation (5), which are −45°, −22.5°, and 0°, as displayed by the green vectors in Figure 4d.

From the above analysis, it is reasonable to claim that twelve phase states can be realized for the +1st harmonic by combining the two methods, as plotted in Figure 4e. It can be seen that the vectors of the other two harmonics are stable during the process. In Note S4 (Supporting Information), more details about the operation are provided. The syntheses of the phases of the 0th and −1st harmonics are also included.

## 3 Simulation and Experiments

### 3.1 Independent Beam Steering at Multiple Harmonics

To validate the capabilities of the STCM to generate multiple‐frequency signals and independently synthesize their amplitudes and phases, nonlinear beam‐steering performances are studied through simulations and experiments using the 2‐bit STCM with 5 × 10 unit cells, as described in Section 5. The metasurface is controlled in columns, and each column contains 5 unit cells. It is normally illuminated by a monochromic signal at 5.5 GHz, which is transmitted by a horn under the *x*‐polarization. More details about the measurement configuration are provided in Note S14 (Supporting Information). To simplify the discussion, we aim to generate three harmonics (−1st, 0th, and +1st orders) and control their scattering performances by using four SSs to comprise the SUD (*S* = 4). The length of each SS is four (*M* = 4), so the length of the SUD *N* = 16. The switching frequency of the controlling signal is set to be 1 MHz, and thus the frequency interval between the harmonics is 62.5 kHz. The experiments include the following three parts.

1. We synthesize the phases of the harmonics by selecting the initial phases φ_initial_ of the BSs. In addition, we tune their amplitude ratio by altering the number of SSs allocated to each harmonic.

**Figure 5:**
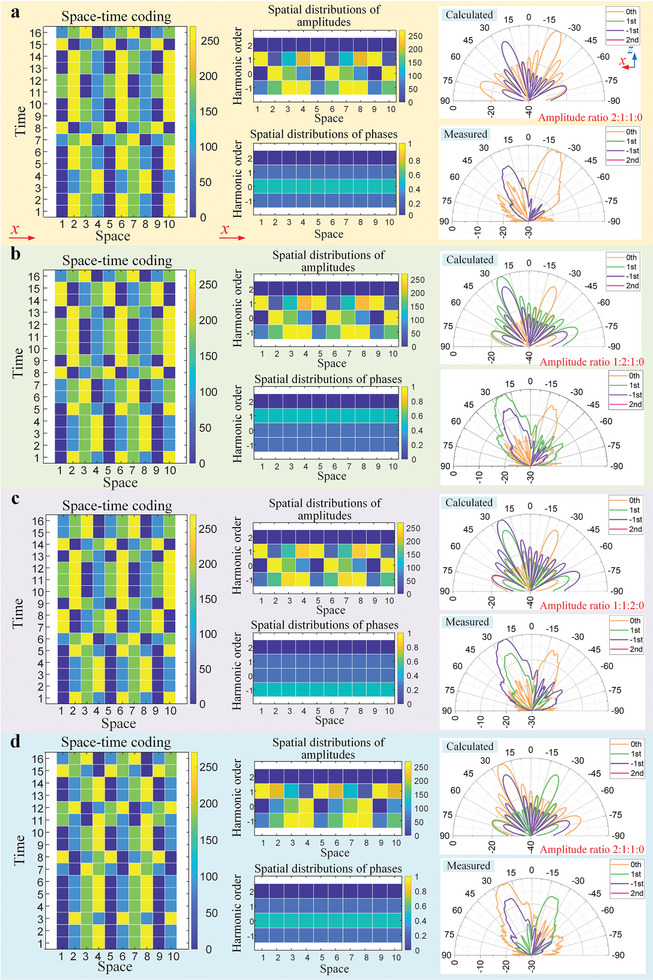
Independent manipulations of the scattering patterns of the −1st, 0th, and +1st harmonics. The harmonic phases are synthesized by selecting the initial phases of the BSs, and their amplitude ratio is tuned by altering the number of BSs for each harmonic. Amplitude ratio of the 0th, +1st, and −1st harmonics are a) 2:1:1, b) 1:2:1, c) 1:1:2, and d) 2:1:1. a–c) Phase gradients of the three harmonics are the same. d) The amplitude ratio is the same as that in figure a, but the phase gradients of the 0th and +1st harmonics are exchanged.

Four beam‐steering cases are shown in **Figure** **5**a–d. In Figure 5a, the numbers of the SSs for the 0th, +1st, and −1st harmonics are 2, 1, and 1, so their magnitude radio is 2:1:1. By selecting BSs with proper initial phases for the columns, different phase gradients along the *x*‐direction are designed for the three harmonics. The 2‐bit space‐time‐coding matrix, which is presented by the phase distribution on the left side of the figure, yields the spatial distributions of equivalent amplitudes and phases of the three harmonics shown in the middle. For the ±1st harmonics, the equivalent phase gradients are −90° per cell; the phase gradient for the 0th harmonic is +90° per cell. Figure 5a plots the calculated and measured 2D scattering patterns of the harmonics, which are in good agreement. The curves of the ±1st harmonics are almost overlapped, with the main lobes pointing to *θ* = 21°. The main lobe of the 0th order is deflected to *θ* = −19.5°. As expected, the magnitude of the 0th harmonic is higher than the others by about 6 dB. In Figure 5b, we set the amplitude ratio to 1:1:2 and keep their phase gradients on the panel unchanged. The results show that the beam directions are the same as those in Figure 5a, but the +1st beam becomes stronger than the other two by approximately 6 dB. It should be noticed that the +1st harmonic amplitude is not precisely doubled, due to the addition of two vectors with an angle between them, which is calculated by *ik*α in Equation (5). The vector angle is −22.5° in this case, resulting in a modulus of 1.96 for the synthesized harmonic vector. In the same manner, in Figure 5c, a similar phenomenon is obtained for −1st harmonic with the magnitude nearly doubled. The deflection angles of these harmonic beams in Figure 5a–c remain constant owing to the unchanged phase gradients, suggesting that the amplitude synthesis does not affect their phases. In Figure 5d, the amplitude ratio is set to be 1:2:1, the same as the one in Figure 5a; in contrast, phase distributions of the 0th and +1st harmonics are exchanged. Comparing the scattering patterns in Figure 5a,d, it is evident that the beam directions of these two harmonics have been interchanged while their magnitudes remain almost unaltered. These phenomena prove that the phase control has nothing to do with the magnitude settings.

1. We conduct a more precise control over the beam direction of the +1st harmonic without affecting its amplitude or the other two harmonics.

As introduced earlier, this is realized by jointly selecting its four BSs and allocating them with different SS index *i*. We keep the index for the −1st harmonic settled and exchange the indices for the 0th and +1st harmonics. This does not influence the phase of the 0th harmonic since the order *k =* 0.

**Figure 6:**
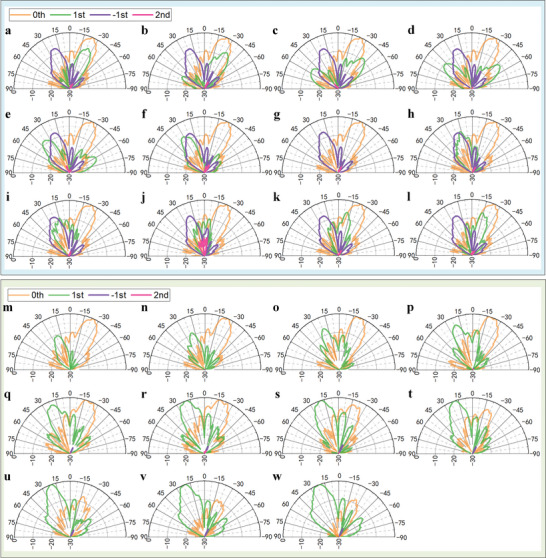
a–l) Measured far‐field scattering patterns of the 0th and ±1st harmonics. It shows that while the main lobe of the +1st harmonic varies in twelve directions, the patterns of the other two harmonics remain unaffected. m–w) Measured far‐field scattering patterns of the 0th and +1st harmonics. Through the joint space‐ and time‐coding strategies, the beam magnitudes are finely adjusted without any influence on their main lobe directions.

Following the principle, twelve scattering patterns of the +1st harmonic with various scanning angles are achieved. The calculated far‐field patterns are presented in Figure S2a in Note S5 (Supporting Information), and the measured ones are displayed in **Figure** **6**a–l, which are in good agreement. The details of the space‐time coding and the equivalent amplitude and phase distributions that lead to these phenomena can be found in Movie S1 (Supporting Information). Figure 6a‐l shows that the beam direction of the +1st harmonic is successively (a) −25°, (b) −32°, (c) −40°, (d) ±49°, (e) +39°, (f) +31°, (g) +21°, (h) +17°, and finally (l) −17°. Due to the blocking effect of the transmitting horn, the main lobes are disrupted in Figure 6i–k. The scanning range covers nearly 100° with an interval of around 8°, suggesting precise control over the phases. Slight magnitude reductions occur to the +1st harmonic when the beam points to large angles, which can be attributed to the increased grating lobes due to the relatively large unit cells. During this process, the patterns of the 0th and −1st harmonics remain fixed. The manipulation of the −1st harmonic is also conducted using the same method, and the calculated and measured results are given in Figure S2b,c in Note S5 (Supporting Information), respectively. These phenomena showcase the ability to finely control the phases of the target harmonic without affecting its amplitude or the other harmonics.

1. We demonstrate the precise manipulations of the beam magnitudes of the 0th and +1st harmonics by utilizing the joint time‐ and space‐domain coding strategy.

As discussed in Section 2.3, in the time domain, the magnitude ratio of the harmonics generated by each column is adjusted by setting the numbers of their BSs. However, the flexibility is very limited especially if the number of SSs is small. Here the degree of freedom is extended by introducing the spatial modulation, and more details about this combined method are explained in Note S6 (Supporting Information).

In this demonstration, the number of SSs is four. For starters, the amplitude ratio in all the columns is 3:1, meaning that the main lobe of the 0th harmonic should be 9.5 dB stronger than the +1st harmonic. We then proceed to successively switch the ratio in each column to 1:3. As the columns with the 1:3 ratio increase, the energy carried by the +1st harmonic is enhanced, and the 0th harmonic beam shrinks gradually. During this process, the phase gradients of the two harmonics remain constant, resulting in the fixed main lobe directions.

Movie S2 (Supporting Information) provides a visual representation of the changes in space‐time coding sequences applied to the columns and the equivalent amplitude and phase distributions of each harmonic. It shows that the equivalent phase gradients remain unchanged throughout the process, while the amplitudes gradually evolve. The calculated far‐field patterns are summarized in Figure S2d of Note S5 (Supporting Information), and the measured ones are displayed in Figure 6m–w. As expected, the main lobe directions remain fixed at ±22°, respectively. As depicted in Figure 6m, the main lobe of the 0th harmonic is approximately 9.5 dB larger than that of the +1st. In Figure 6n through w, the 0th harmonic gradually weakens while the +1st harmonic becomes stronger. When half of the ten columns hold the amplitude ratio of 1:3, the magnitudes of the two harmonics are equal, as shown in Figure 6r. Finally, in Figure 6w, the +1st harmonic beam is nearly 9.5 dB higher than the other.

In Note S7 (Supporting Information), we present the independent manipulations of four harmonic scattering beams using five SSs, strongly validating the flexibility and independent control over the amplitudes and phases of these harmonics.

### 3.2 Simultaneous and Independent Transmissions for Modulated and Unmodulated Signals

As an illustrative application, we employ the STCM as a hybrid wireless transmitter for simultaneous transmissions of modulated and unmodulated signals at various harmonics. Two modulation schemes, BFSK and QPSK, are realized respectively. The *x*‐polarized horn is used to excite the metasurface at 5.5 GHz. A pair of *y*‐polarized horns are utilized as the receiving antennas. One is named Horn B, used to receive the unmodulated 0th harmonic energy deflected by the metasurface and inject it into a signal analyzer. The other is named Horn A, used to receive the modulated signal at +1st or −1st harmonic frequencies. A software‐defined radio (SDR) reconfigurable device (TQTT B210) is connected to it, which functions to recover the transmitted data. More details about the experiment setup are presented in Note S14 (Supporting Information), and the system schematic for the wireless communication is presented in Note S8 (Supporting Information).

1. Experiment with the BFSK communication and unmodulated beam deflection.

Three harmonics (0th and ±1st) are utilized. The ±1st harmonics are a pair of discrete frequencies for the BFSK modulation, with their amplitudes dynamically regulated to present opposite coding states. In addition, the phases of these two harmonics are designed to form a phase gradient on the panel, allowing the signals to be sent in a predesigned direction (+22.9°). Concurrently, the 0th harmonic signal without modulation is solely tilted to another direction (−22.9°), which is achieved by setting its phase gradient on the metasurface. These tasks are implemented by using three SSs, two of which are utilized to generate the 0th harmonic, while the remaining one is utilized to generate either the +1st or −1st harmonic. The switching frequency of the controlling signal is set to 1 MHz. Because the code length is 12, the frequency interval between the harmonics is 83.3 kHz. The sampling rate is 2 M Sa s^−1^. The data rate is 83.3 Kbps. The fundamental frequency generated by the signal generator is ‐23 dBm.

**Figure 7:**
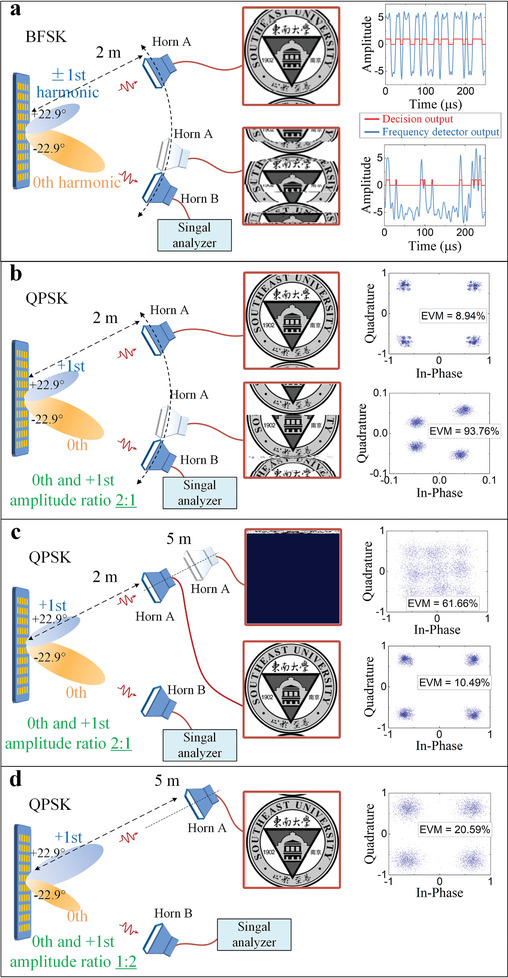
Measured results in the simultaneous modulated and unmodulated signal transmissions. a) BFSK modulation is realized using the ±1st harmonics, and the signal is directed to +22.9°. The unmodulated signal at the 0th harmonic is directed to −22.9°. Only when Horn A is in the correct direction can the information be well recovered. b–d) QPSK modulations are realized at the +1st harmonic, and the signal is directed to +22.9°. The unmodulated signal at the 0th harmonic is directed to −22.9°. b) The amplitude ratio of the unmodulated and modulated signals is 2:1. The distance between Horn A and the STCM is 2 m. If Horn A deviates from the right direction, the data cannot be satisfactorily recovered. c) When Horn A moves further from the metasurface from 2 to 5 m, the data transmission deteriorates. d) The data transmission recovers when the modulated signal at the +1st harmonic is enhanced by switching the amplitude ratio to 1:2.

Experimental results are presented in **Figure** **7**a. When Horn A is positioned in the correct direction, the picture is well recovered, and the receiving performance is confirmed by the temporal frequency detector and decision outputs offered in the upper right inset. The frequencies of the signal are precisely detected, and the codings are accurately demapped. However, if Horn A is moved away to other directions, the picture cannot be received, which is also evident in the results of the frequency detector and decision output. Movie S3 (Supporting Information) shows a video of the change in the communication results as Horn A moves. When Horn B is positioned in the correct direction of −23°, the received 0th harmonic is at its maximum (−66.9 dBm); however, if it deviates from the preset direction, the received energy is declined. The far‐field scattering pattern of the 0th harmonic is presented in Figure S6a in Note S9 (Supporting Information). Movie S4 (Supporting Information) shows the change of the received 0th amplitude as Horn B moves.

1. Experiment with the QPSK communication and unmodulated beam deflection.

Two harmonics (0th and +1st) are utilized. The +1st harmonic is used for the QPSK modulation in the direction of +22.9°, and the 0th harmonic is unmodulated and tilted to a direction of −22.9°. Three SSs are employed, with two used for the 0th harmonic and the other for the +1st harmonic. The switching frequency of the controlling signal, the frequency interval, and the sampling rate are the same as those in the BFSK experiment. The data rate is 166.7 Kbps.

The results are shown in Figure 7b when the fundamental frequency is 20 dBm. When Horn A is placed in the correct direction of +23°, the data is successfully received, and the picture is adequately restored. The constellation diagram, shown in the upper right inset, is of high quality, with an error vector magnitude (EVM) of 8.94%. However, as the horn moves away to other directions, the constellation diagram deteriorates, as shown in Movie S5 (Supporting Information). As an example, Figure 7b shows the results when Horn A is placed in the direction of about −23°. It is observed that the picture is distorted, and the constellation diagram is cluttered with an EVM of 93.76%. The change of EVM during this process is presented in Figure S7a in Note S10 (Supporting Information). The 0th harmonic received by Horn B achieves its maximum −22.4 dBm when the horn is located in the correct direction of −23°. Movie S6 (Supporting Information) shows the variation of the received 0th harmonic as Horn B moves. The far‐field scattering pattern of the 0th harmonic is presented in Figure S6b in Note S9 (Supporting Information).

To showcase the effectiveness of the amplitude control in the QPSK experiment, we keep Horn A in the correct direction and increase the distance between it and the STCM from 2 to 5 m. The power of the fundamental frequency is set to −16.5 dBm. As shown in Figure 7c, when the distance is 2 m, a high‐quality picture is received, and the constellation diagram is clear with an EVM of 10.49%. While the antenna is moved further, the constellation diagram gradually deteriorates with the increasing EVM, as presented in Figure S7b in Note S10 (Supporting Information). When the distance is 5 m, the constellation diagram becomes cluttered (EVM = 61.66%), and the information cannot be recovered, as shown in Figure 7c. Then, we adjust the amplitude ratio of the 0th and +1st harmonics from 2:1 to 1:2. This results in an approximate 6 dB enhancement of the QPSK signal. As displayed in Figure 7d, the constellation diagram improves significantly. The EVM drops back to 20.59%, and the picture is satisfactorily received again. The 0th harmonic magnitude received by Horn B is reduced by around 6 dB. Movie S7 (Supporting Information) provides a video illustrating the communication result, including the changes that occur when Horn A moves further and the improvement when the amplitude ratio is switched.

In the above proof‐of‐concept investigations, the disentanglement of the harmonics is adequately demonstrated by using the 2‐bit STCM. It is important to note that using multi‐bit metasurfaces and incorporating more SSs would enable the exploitation of additional harmonics with more precise controls for the execution of SFDM tasks. On the other hand, longer coding sequences necessitate a higher switching speed of the controlling signal and sampling rate, which requires a more advanced FPGA platform and driving circuits. Furthermore, high‐performance devices for demodulation and baseband signal processing are also necessary. In the next step of our work, we will utilize commercial controlling platforms and SDR USRP devices from National Instruments Corp and try to apply the proposed technique to other FDM applications such as target detection, imaging, and so on.

## 4 Conclusion

To summarize, we presented an STCM that is capable of disentangling the amplitudes and phases of multiple harmonics through an innovative time‐modulated coding strategy. Different from previous studies, the proposed method introduced the temporal BSs that are responsible for the harmonic generation in the frequency domain. By taking advantage of this, the mechanism behind the harmonic synthesis was theoretically explained, and the coding sequences were efficiently designed via analytical approaches. Combined with the full use of spatial modulations on the metasurface, scattering beams of multiple harmonics were manipulated independently, which were validated by simulations and measurements. To further prove the advantages of the proposal, the SFDM wireless experiments involving modulated and unmodulated signal transmissions were successfully performed. With the ability to generate multiple harmonics with disentangled amplitudes and phases, the proposed STCM has shown great potential in various SFDM scenarios, such as radar detection, wireless communication, simultaneous information and power transfers, and so on.

## 5 Experimental Section

### Methods: Metasurface Design

**Figure 8:**
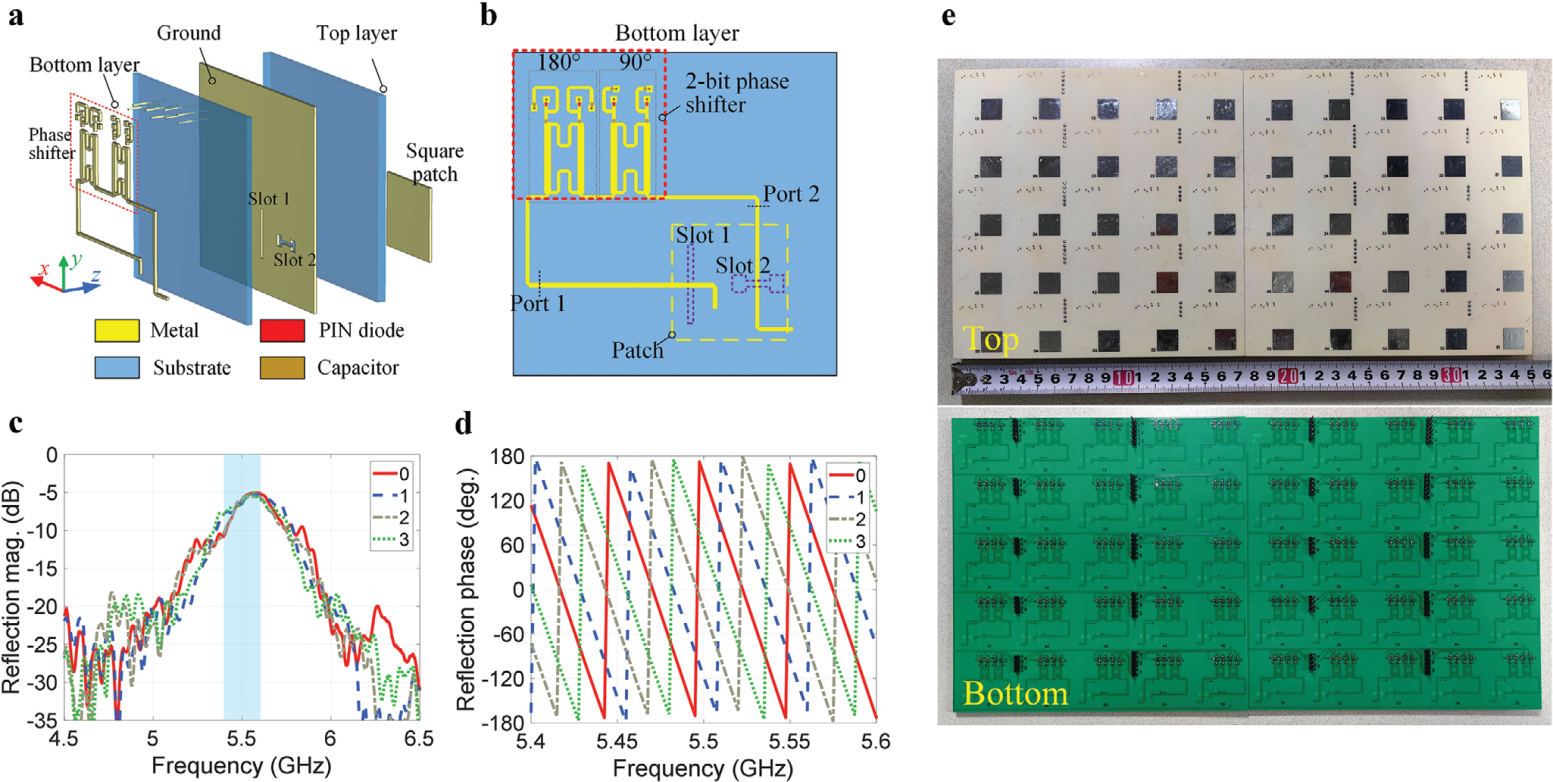
The 2‐bit reconfigurable metasurface. a) 3D view of the unit cell of the STCM. b) Metallic pattern on the bottom layer of the unit cell, where the 2‐bit phase shifter is located. c) Measured reflection magnitudes of the STCM working at the four coding states. d) Measured reflection phases of the STCM working at the four coding states. e) Pictures of the STCM prototype.

As presented earlier, the number of controllable harmonics depends on the number of coding states of STCM. In this work, the 2‐bit reflective STCM is designed to prove the proposed time‐varying coding strategy. The structure of the metasurface unit cell is shown in **Figure** **8**a. It is a commonly used receiver‐transmitter framework of reconfigurable metasurfaces, holding advantages such as low profile, stable reflection magnitudes, and high integration. It includes three metallic layers that are separated by two substrates. On the top layer is a square patch that can work under the orthogonal polarization states. The second layer is called the ground layer, which is a metallic plate with two slots, functioning for EM coupling between the top and bottom layers. As displayed in Figure 8b, an electrically reconfigurable phase shifter is located on the bottom layer. When *x*‐polarized EM waves from the space impinge on the top patch along the *z*‐axis, the EM energy is received and coupled downwards into guided waves through Slot 1. The waves are then guided by the microstrip on the bottom layer and injected into the phase shifter. After the phase‐shifting behavior, the guided waves are coupled to the patch through Slot 2, and radiate out into space normally as *y*‐polarized waves. Because the two slots are orthogonally located beneath the radiating patch, the incident and radiated waves are cross‐polarized with a strong isolation. More details of the phase shifter and radiators are presented in Notes S11 and S12 (Supporting Information), respectively.

To validate the design of the metasurface, a prototype array with 5 × 10 unit cells is fabricated using the printed‐circuit‐board technology, whose picture is shown in Figure 8e. It measures 175 mm × 350 mm, and the thickness is 1.8 mm. The biasing wires of the phase shifters of the unit cells are connected to an FPGA platform based on Cyclone IV E (EP4CE10F17C8) through a custom‐built drive circuit, and every single unit cell can be controlled individually. The FPGA is preloaded with a code that describes the controlling commands.

The reflection properties of the array are first measured using the experiment configuration provided in Note S14 (Supporting Information). All the unit cells are controlled by the same signal, so their reflection phases are identical. Figure 8c,d displays the reflection magnitudes and phases, respectively. The magnitude curves of the four coding states are almost identical within the bandwidth of interest. The reflection magnitudes at 5.55 GHz are around −5.0 dB. The loss is attributed to the insertion loss of the hybrid reflection‐type phase shifters. As presented in Note S11 (Supporting Information), the measured insertion loss of the phase shifter is 4.4 dB. Figure 8d shows that the reflection phases undergo stable changes of 90° between the curves from 5.4 to 5.6 GHz, indicating a satisfactory phase‐shifting performance. The coding states “0, 1, 2, 3” correspond to the phase states “0°, 90°, 180°, 270°.” If the reflection magnitudes are normalized, the coding states can be also presented as “1, j, −1, ‐j” in the complex plane. Before the nonlinear experiments using this metasurface, its anomalous reflection performances at 5.5 GHz under the control of static coding sequences are measured. The theories and measured far‐field scattering patterns can be found in Note S13 (Supporting Information).

## Conflict of Interest

The authors declare no conflict of interest.

## Supporting information

Supporting Information

Supplemental Movie 1

Supplemental Movie 2

Supplemental Movie 3

Supplemental Movie 4

Supplemental Movie 5

Supplemental Movie 6

Supplemental Movie 7

## Data Availability

The data that support the findings of this study are available from the corresponding author upon reasonable request.
