# Supplementary material for: Fully Breaking Entanglement of Multiple Harmonics for Space‐ and Frequency‐Division Multiplexing Wireless Applications via Space‐Time‐Coding Metasurface
Source: Adv Sci (Weinh). 2024 Jul 4;11(34):2404558. doi: 10.1002/advs.202404558 (PMC11425898; doi:10.1002/advs.202404558)
Supplement: Supplementary file 1 — Supporting Information [file ADVS-11-2404558-s008.docx]

Supporting Information

Fully Breaking Entanglement of Multiple Harmonics for Space- and Frequency-Division Multiplexing Wireless Applications via Space-Time-Coding Metasurface

Zhangjie Luo, Zhiming Zhang, Junwei Tai, Lei Zhang, Chenglong Gao, Hui Feng Ma, Wei Xiang Jiang, Qiang Cheng*, and Tie Jun Cui*

**S1. Comparisons between this work and the previous works**

**S2. Limitations of the length of sub-sequences and the number of harmonics that can be controlled**

**S3. Derivation of Equation (3)**

**S4. Independent phase syntheses of the -1st, 0th, and +1st harmonics**

**S5. Calculated and measured beam-steering phenomena of the harmonics using the STCM**

**S6. Precise control over the amplitude ratio of the 0th and +1st harmonics through time- and space-domain strategies**

**S7. Calculated beam-steering phenomena of four harmonics using the STCM**

**S8. System schematics of the transmitter and receiver in the wireless communication experiments**

**S9. Far-field scattering patterns of the 0th harmonic in the wireless communication experiments**

**S10. Error Vector Magnitude (EVM) in the QPSK wireless communication experiment**

**S11. Quadrature hybrid reflection-type phase shifter**

**S12. Performances of the radiators of the metasurface unit cell**

**S13. Anomalous reflection performances of the metasurface**

**S14. Experimental setups**

## S1. Comparisons between this work and the previous works

Table S1. Comparisons between our design with others in the literature.

| Reference | [1] | [2] | [3] | [4] | [5] | **This work** |
| --- | --- | --- | --- | --- | --- | --- |
| Number of independently controllable harmonics | 1 | 2 | 1 for each partition | 7 | $2^{q}$ (for q-bit metasurface) | $2^{q}$ **(for q-bit metasurface)** |
| Disentangled amplitude and phase | Amplitude and phase | Amplitude and phase | Amplitude and phase | Phase only | Phase only | **Amplitude and phase** |
| Unit cells used for harmonic manipulation | All | All | Partial | All | All | **All** |
| Analytical | Yes | Yes | Yes | No | Yes | **Yes** |
| Tunable components | Varactor | Varactor | Varactor | PIN diode | PIN diode | **PIN diode** |
| Hardware | FPGA, DAC, and amplifier | FPGA, DAC, and amplifier | FPGA, DAC, and amplifier | FPGA | FPGA | **FPGA and current driving circuit** |
| Cost | High | High | High | Low | Low | **Low** |

The advantages of the proposed space-time-coding metasurface (STCM) can be summarized as follows.

(1) The number of harmonics that can be independently controlled in this work is $2^{q}$, which is the tunable phase states of the STCM. Here, *q*=2, so the disentangled harmonic number is 4. This number was 1 and 2 in Refs. [1] and [2], respectively. In Ref. [3], the disentangled harmonic number depended on the number of the partitions. As the scale of the metasurface increases, more partitions could be made to increase the harmonics.

(2) Both the amplitude and phases of the harmonics are disentangled in this work and Refs. [1] through [3]. However, only phases were disentangled in Refs. [4] and [5].

(3) The time-coding strategy in this work is analytical, which means that optimization process is not required for designing the controlling sequence. This makes the design more effective.

(4) Different from the study in [3], the proposed metasurface utilizes all the unit cells to generate target harmonics, making full use of its aperture for anomalous deflections of their beams. This maximizes its potential for space-division multiplexing. In [3], however, each harmonic was only manipulated by its partition, not a whole aperture, so the ability for the beam manipulation was reduced.

(5) The proposed STCM employs PIN diodes as the tuning components, which only needs a low-cost field programmable gate array (FPGA) platform based on Cyclone IV E (EP4CE10F17C8) and a custom-built drive circuit. In comparison, the STCM in Refs. [1] through [3] employed varactors as the tuning components, which required expensive digital-analog converters (DACs) and amplifiers to convert digital signals from FPGA to biasing voltages.

## S2. Limitations of the length of sub-sequences and the number of harmonics that can be controlled

Take a *q*-bit space-time coding digital metasurface (STCM) with $2^{q}$ coding states as an example. To prove that the length of sub-sequences and the number of harmonics that can be controlled are limited by the number of coding states $2^{q}$, we assume that the length of sub-sequences is *M’*. The required discrete Fourier transform (DFT) sequences are orthogonal in the frequency domain, which can be exhaustively listed as

, , …, ,

.

In other words, the required DFT sequences can be expressed as

(S1)

where $k'$ is the harmonic order, and $0\leq k'\leq M'$. For each DFT sequence, there are *M’* values for the phase *φ*, which are

, ,, , .

Then, the inverse DFT (IDFT) of $\text{X(}k)$ is

. (S2)

$\text{x(}n)$ the temporal sequence that would be applied to the STCM, which is the basic sequence (BS) in the main text of this paper. From Equation (S2), we can see that the phase of the sequence is . Because the phase states that can be provided by the STCM is $2\pi\frac{v}{M}$ ($0\leq v\leq M-1$), the following equations should be satisfied.

. (S3)

Therefore, the maximum value of *M’* is *M*, and the phase of the BS is also limited by *M*. As indicated in the main text, the number of harmonics that can be controlled is limited by the sub-sequence length *M’*. In this study, we let *M’*= *M*=$2^{q}$.

## S3. Derivation of Equation (3)

$\begin{matrix} F_{i}(k)=\sum_{n=0}^{N-1} G_{i}(n)\exp(-j\frac{2kn\pi}{N}) \\ \text{ }=\sum_{m=0}^{M-1} U_{i}(m)\exp(-j2\pi k\frac{S\cdot m+i}{N}) \\ \text{ }=\sum_{m=0}^{M-1} U_{i}(m)\exp(-j2\pi k\frac{m}{M})\exp(-j2\pi\frac{ik}{N}) \\ \text{ }=\sum_{m=0}^{M-1} U_{i}(m)\exp(-j2\pi(M\cdot s+r)\frac{m}{M})\exp(-j2\pi\frac{ik}{N}) \\ \text{ }=\sum_{m=0}^{M-1} U_{i}(m)\exp(-j2\pi r\frac{m}{M})\exp(-j2\pi\frac{ik}{N}) \\ \text{ }=P_{i}(r)\exp(-j2\pi\frac{ik}{N}) \\ \text{ =}P_{i}(k\mathrm{mod} M)\exp(-j2\pi\frac{ik}{N}) \end{matrix}$ (S4)

## S4. Independent phase syntheses of the -1st, 0th, and +1st harmonics


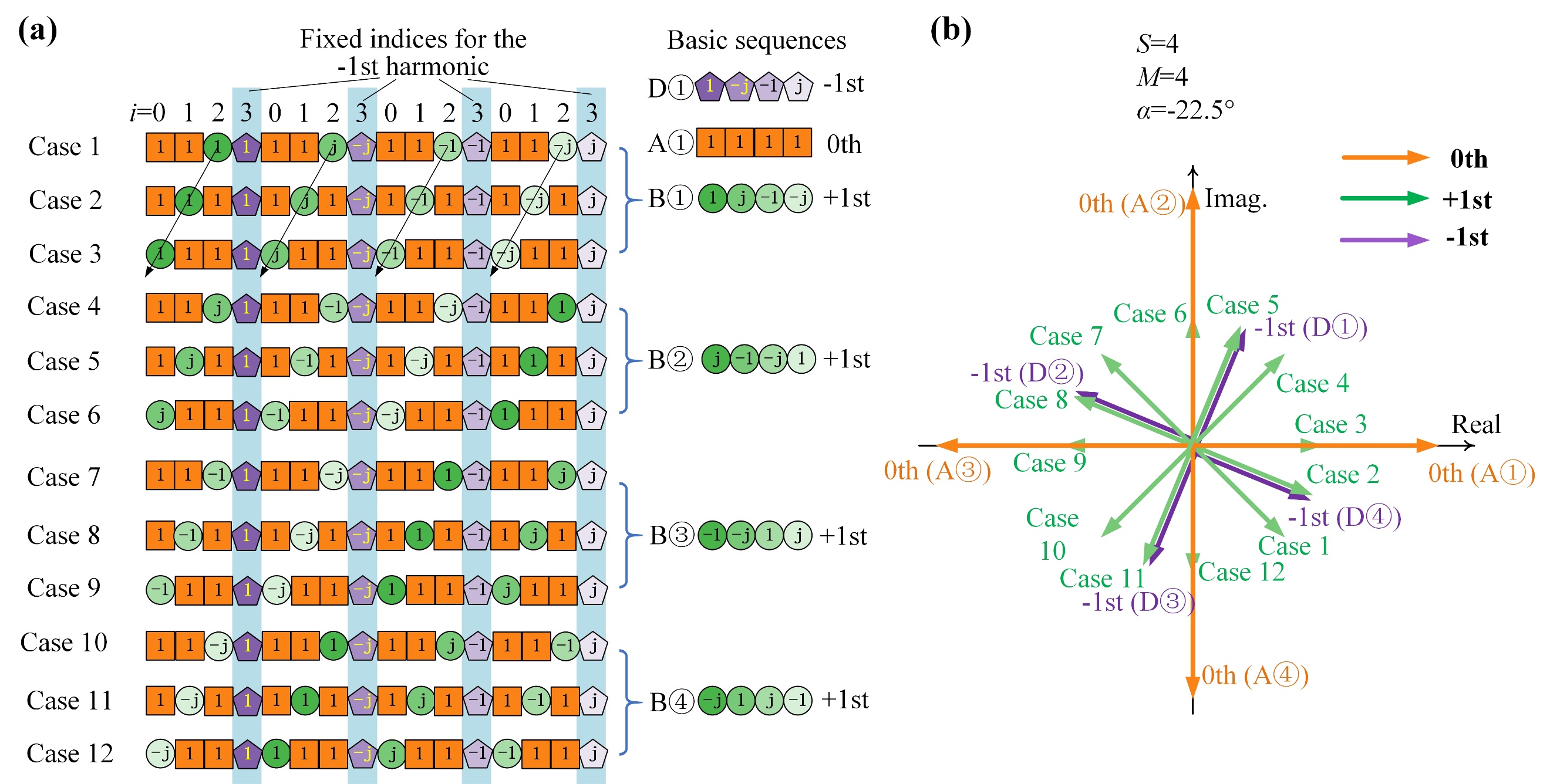


**Figure S1.** Independent phase syntheses of the -1st, 0th, and +1st harmonics. a) Twelve SUDs that yield twelve phases of the +1st harmonic. This is realized by combining the two methods for synthesizing the phase of the +1st harmonic, that is, BS selection and index switching. This does not impact the phase of the 0th harmonic because its order *k* is 0. b) Vectors of the three harmonics in the complex plane. The vector of the +1st harmonic rotates counterclockwise with a constant amplitude, indicating the twelve phase states. Amplitudes and phases of the 0th and -1st harmonics remain unaffected. To individually adjust the phases of the 0th and -1st harmonics, we can change their BSs with different initial phases, respectively.

**Figure S1** illustrates an example of independently manipulating the phases of three harmonics (-1st, 0th, and +1st orders) without affecting their amplitudes. For the +1 harmonic, this is achieved by combining the two methods introduced in the main text, that is, BS selection and index switching. For the other two harmonics, this is realized by the BS selection.

Figure S1 shows the twelve cases of SUDs to finely tune the phase of the +1st harmonic while keeping the phases of the 0th and -1st harmonics and the amplitudes of the three harmonics unchanged. The number of sub-sequences is four (*S*=4), and the SS length is four (*M*=4), so the length of the SUD is sixteen (*N*=16). Two BSs A①[1, 1, 1, 1] are included to generate the 0th harmonic. One BS D①[1, -j, -1, j] is selected to generate the -1st harmonic. For the +1st harmonic, its BSs B①, B②, B③, or B④ are ready to be chosen. Because the numbers of BSs are determined, the amplitude ratio is fixed.

The SS index allocated to D① is fixed to be 3, so the phase of the -1st harmonic is constant, that is, . Since the order *k* is 0 for the 0th harmonic, its phase keeps 0° if we switch the indices of its BSs with that of the +1st harmonic.

As shown in Figure S1a, in cases 1 through 3, the BS of the +1st harmonic is B①[1, j, -1, -j], whose initial phase is 0°. In case 1, the index for it is 2, so its phase . By changing the index from 2 through 0, the phase varies from -45° through 0° with an interval of -22.5°, as shown by the green vector in the complex plane in Figure S1b. In cases 4 through 6, we change the BS of the +1st harmonic to B②[j, -1, -j, 1], whose initial phase is 90°. Similarly, by changing its index, its phase varies successively from 45°, 67.5°, through 90°. In this way, in cases from 7 through 12, we realize six more phases for the +1st harmonic from 135° to 270°. As presented in Figure S1b by the green vector, the +1st harmonic vector rotates counterclockwise with a constant amplitude. During this process, the 0th and -1st harmonic vectors remain unchanged.

Figure S1b also illustrates the syntheses of the phases of the 0th and -1st harmonics by changing their initial phases, which is implemented by changing their BSs. Specifically, when we change the BS of the 0th harmonic successively to A②, A③, and A④, its phase varies to 90°, 180°, and 270°, as shown by the orange vector in Figure S1b. In the same manner, by changing the BS of the -1st harmonic to B②, B③, and B④, its phase can be adjusted to 157.5°, 247.5°, and 337.5°, successively, as illustrated by the purple vector in the figure. It can be observed that the phase changes of each harmonic are independent of one another.

## S5. Calculated and measured beam-steering phenomena of the harmonics using the STCM


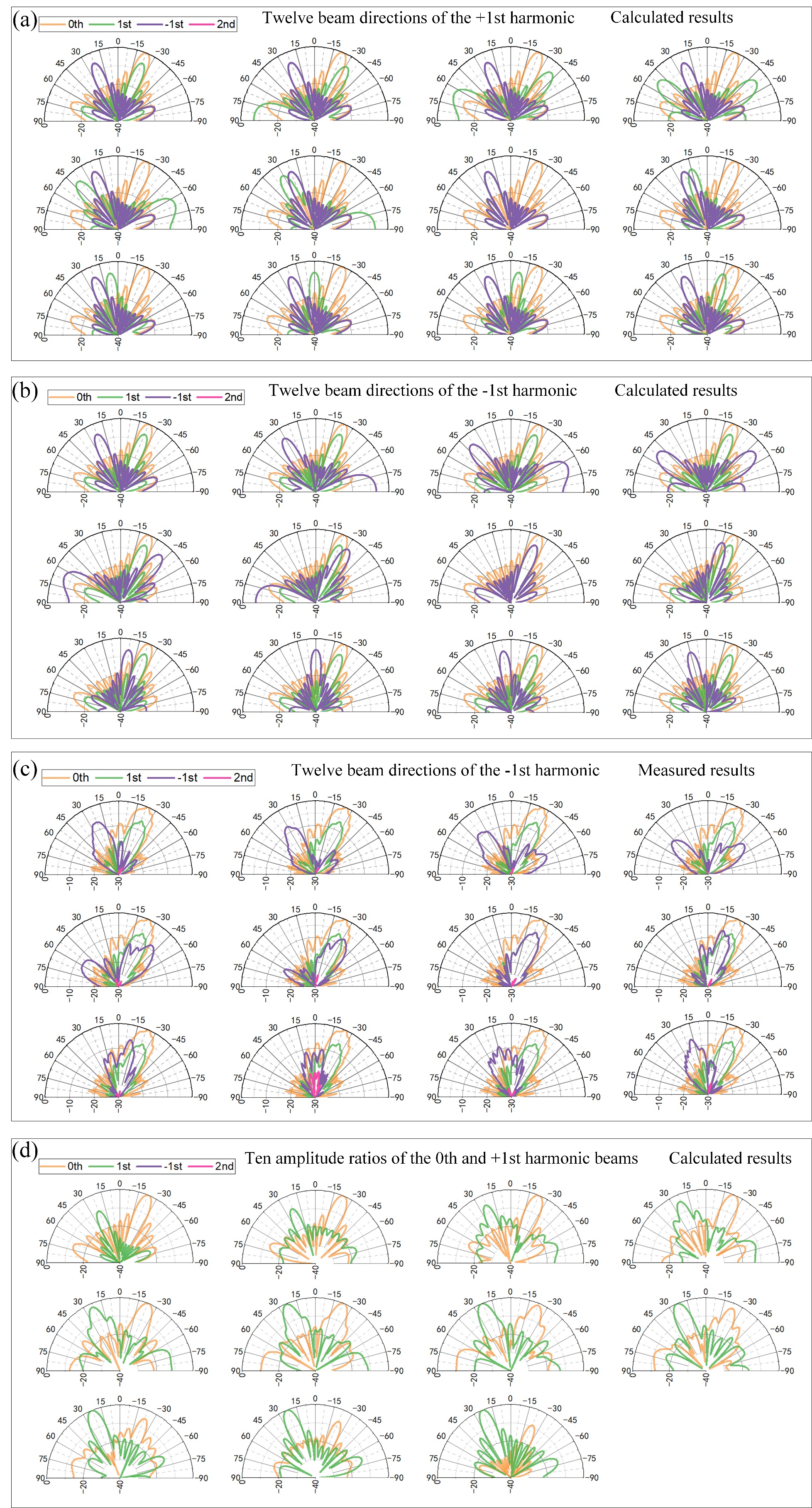


**Figure S2.** Calculated and measured far-field scattering patterns of the harmonics. (a) Calculated twelve beam directions of the +1st harmonic. The main lobes of the 0th and -1st harmonics remain unchanged. (b) (c) Calculated and measured twelve beam directions of the -1st harmonic. The main lobes of 0th and +1st harmonics remain unchanged. (d) Calculated scattering patterns of the 0th and +1st harmonics. While their amplitude ratio is precisely adjusted by the joint time- and space-domain coding strategies, the directions of their main lobes remain constant.

## S6. Precise control over the amplitude ratio of the 0th and +1st harmonics through time- and space-domain strategies


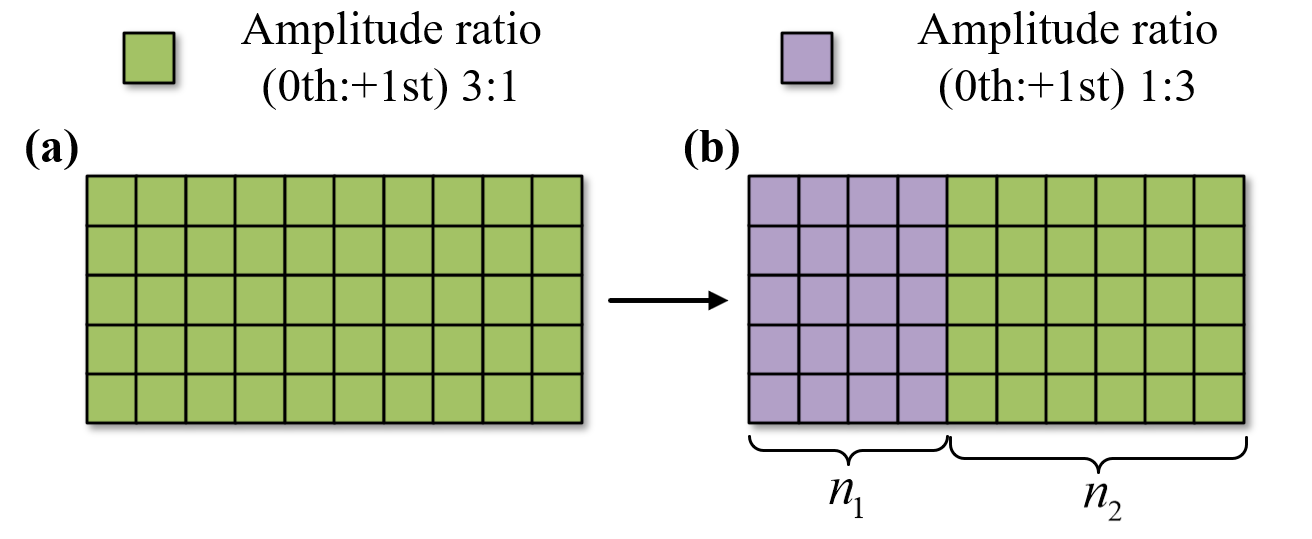


**Figure S3**. Time- and space-domain strategy for tuning the amplitude ratio of the two harmonics.

We take an array of several columns of unit cells to explain the precise control over the amplitude ratio of the 0th and +1st harmonics, which is implemented through the joint time- and space-domain strategy. Here the number of the sub-sequences is set to four.

As shown in Figure S3, there are two kinds of unit cells in the array. The purple cells mean that the amplitude ratio of the 0th and +1st harmonics they generate is *a*_1_:*b*_1_, and the amplitude ratio of the green cells is *a*_2_:*b*_2_, which are set by setting their BSs in the time domain. The numbers of the columns with the purple and green cells are *n*_1_ and *n*_2_, respectively. So, the amplitude ratio provided by the whole array is

$\frac{A_{0th}}{A_{+1st}}=\sqrt{\frac{a_{1}^{2}n_{1}+a_{2}^{2}n_{2}}{b_{1}^{2}n_{1}+b_{2}^{2}n_{2}}}$. (S5)

Therefore, by adjusting the values of *n*_1_ and *n*_2_, the ratio can be precisely tuned. It should be noticed that the phases of each harmonic keep stable when their amplitudes are changed, which guarantees the stability of the directions of the scattered harmonics.

In the experiment in the main text, the number of columns in the STCM is 10, that is, *n*_1_+*n*_2_=10. In addition, the amplitude ratios *a*_1_:*b*_1_=1:3 and *a*_2_:*b*_2_=3:1. Therefore, 11 magnitude ratios are achieved in the beam-scanning experiment, as shown in Figure S2d here and Figure 6m-w in the main text.

## S7. Calculated beam-steering phenomena of four harmonics using the STCM


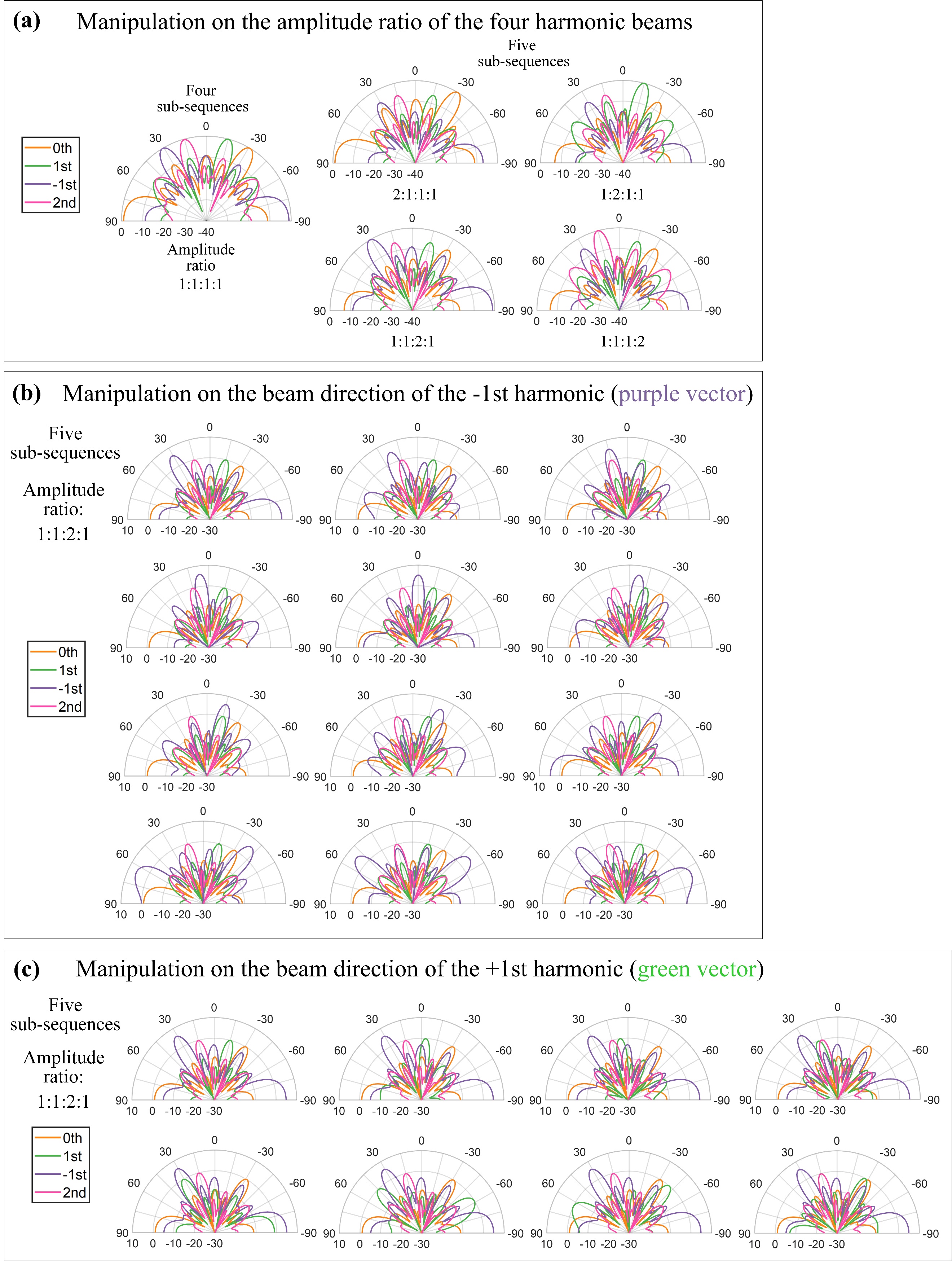


**Figure S4.** Calculated beam-steering phenomena of four harmonics using five sub-sequences. (a) Four sub-sequences are used to control the four harmonics, whose main lobe magnitudes are the same, each pointing to different directions of -31.3°, -15.1°, 31.3°, and 15.1°, respectively. Adding an extra sub-sequence for any harmonic results in its magnitude stronger than the others by almost 6 dB, while the beam directions remain unchanged. (b) Five sub-sequences are employed to generate four harmonics, two of which are assigned to the -1st harmonic, resulting in an amplitude ratio is 1:1:2:1. The main lobe of the -1st harmonic is altered with twelve different directions: 31.3°, 22.9°, 15.1°, 7.5°, 0°, -7.5°, -15.1°, -22.6°, -31.3°, -40.5°, ±51.2°, and 40.5°. To better illustrate the stable scattering patterns of the 0th, 1st, and 2nd harmonics, all the patterns are normalized by the maximum value of these three. The maximum value of the -1st varies slightly, which can be explained by the vector superposition in the complex plane. (c) The number of sub-sequences and the amplitude ratio are the same as in (b). The main lobe of the -1st harmonic is altered with eight different directions: -15.1°, -4.0°, 7.4°, 19.2°, 31.3°, 45.5°, -41.5°, and -27.3°. The magnitudes of all the beams remain unchanged, and the beam directions of the other three harmonics are unaltered, suggesting that the phase change of the -1st harmonic does not impact its magnitude or other harmonics.

## S8. System schematics of the transmitter and receiver in the wireless communication experiments


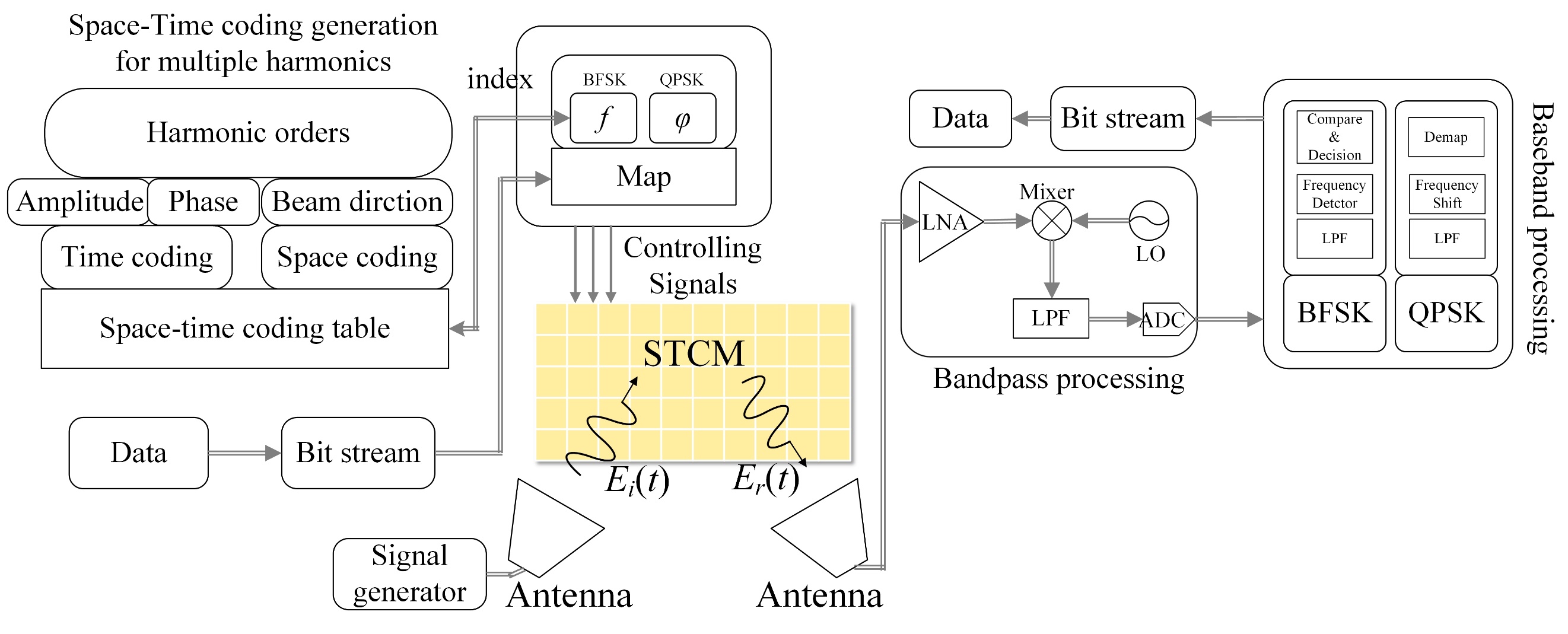


**Figure S5.** Schematics of the transmitter and receiver in the wireless communication experiments

On the transmitter side, based on the proposed time-coding strategy for specific harmonic orders, a space-time-coding table is created for binary frequency-shift keying (BFSK) and quadrature phase shift keying (QPSK) modulations. The information, which is the logo of Southeast University, is decoded into a binary bit stream to the baseband module and then mapped to the corresponding time coding for specific modulation schemes. For BFSK in this experiment, the coding “1” is mapped to the +1st harmonic, and “0” is mapped to the -1st harmonic. For QPSK, the four codings are mapped to the four phase states of the +1st harmonic, respectively. All the signals are steered in certain directions by integrating the space-coding strategy and thus creating specific phase gradients on the metasurface. After the controlling codes are prepared, they are preloaded in the field programmable gate array (FPGA) platform.

On the receiving side, the received RF signal is first downconverted to a baseband digital signal using the software-defined radio (SDR) reconfigurable device (TQTT B210). The baseband signal is then transmitted to the baseband demodulation platform, GNU Radio 3.10, on the laptop computer through a USB 3.0 interface. The signals are firstly filtered by a digital filter to remove out-of-band signals, and then they are demodulated using the appropriate method. In the case of BFSK modulation, the signal is passed through a frequency detector module, which outputs positive or negative values for the +1st or -1st harmonic, respectively. After that, the decision maker assigns a logic level of “1” to signals greater than 0 and a logic level of “0” to signals less than 0. This results in an information bit stream that represents the original data that is transmitted. For QPSK modulation, the signal is processed through a digital frequency shift module to shift the spectrum center to zero, and then a QPSK constellation demapping is performed to obtain the information bit stream. Finally, the bit stream is decoded into the information.

## S9. Far-field scattering patterns of the 0th harmonic in the wireless communication experiments


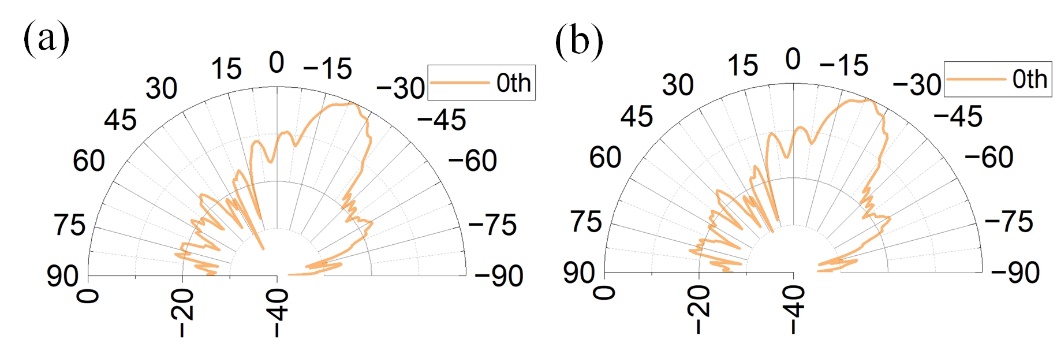


**Figure S6.** Far-field scattering patterns of the 0th harmonic in the wireless communication and beam deflection experiments. Not modulated for communication, this harmonic is only deflected in the preset direction of -23°. (a) Together with the BFSK modulation. (b) Together with the QPSK modulation.

## S10. Error Vector Magnitude (EVM) in the QPSK wireless communication experiment


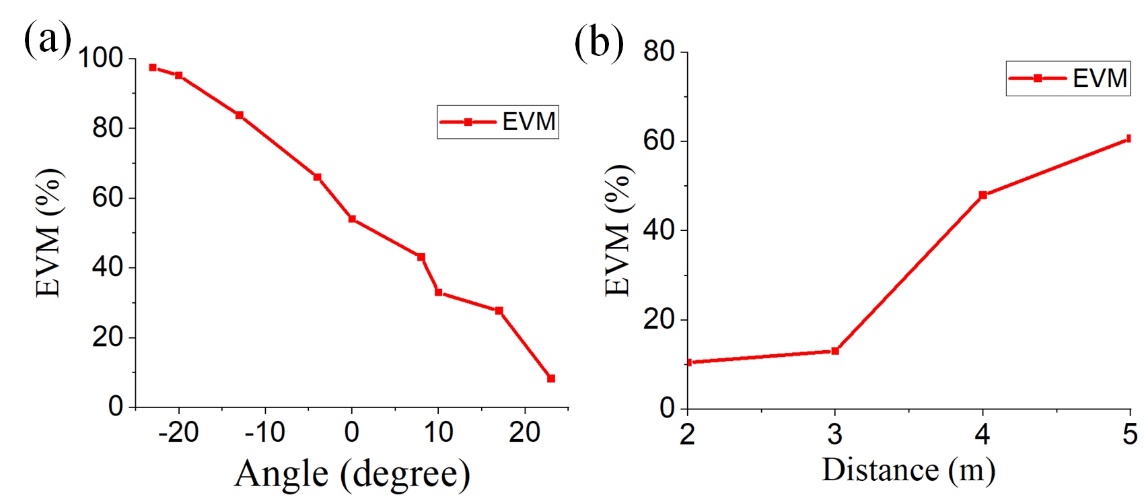


**Figure S7.** (a) The EVM versus the direction of Horn A. The distance between the antenna and the STCM is 2 m. (b) The EVM versus the distance between Horn A and the STCM. Horn A is placed in the correct direction of about 23°.

## S11. Quadrature hybrid reflection-type phase shifter

Each unit cell of the metasurface contains two reflection-type phase shifters (RTPSs) for the 2-bit phase shifting. As plotted in Figure S8a, each RTPS is composed of a hybrid coupler with the through and coupled ports connected with two identical reflective loads. Each load comprises a PIN diode and a microstrip in parallel, which are connected to the ground layer through the metallic blind vias. Direct-current (DC) biasing lines for the diodes are connected to the load parts through 39-nH inductors, which are utilized to choke RF currents; here they are not shown in the figure for the sake of brevity. By changing the DC voltages across the two diodes of an RTPS simultaneously, their working states are switched between ON or OFF, and thus a phase shift of *α* or 0° can be obtained. The phase shift *α* depends on the length of the microstrip that is parallel to the diode. In this work, the 90° and 180° RTPSs are designed and cascaded; thus, four phase shifts, or 2-bit coding states, are achieved. To shrink the size of the RTPSs, the 50-Ω microstrips are folded as meander lines. The capacitors *C* are 47 pF, and the PIN diode is MADP-000907-14020x from MACOM Technology.

The 2-bit phase shifter is simulated using the field-circuit co-simulations in CST. In the field simulations for the microstrip structures, the capacitors are set as the lumped unit cells, and discrete ports are set for the diode connections. Two wave ports are set as the input and output ports of the coupler for EM excitations. After that, in the circuit schematic simulations, external ports 1 and 2 are connected to the input and output ports of the coupler, respectively; scattering parameters (S-parameters) of the PIN diode under the 0-V reverse bias and 10-mA forward bias conditions (s2p files), which are provided by the manufacturer, are connected to the discrete ports. In this way, the reflection coefficient at the input port and transmission coefficients between the input and output ports are obtained. Here the four coding states (states 0, 1, 2, 3) are defined by the four transmission phase shifts with a 90° interval, which are switched by setting the working states of the PIN diodes.

The phase shifter is fabricated, whose picture is shown in Figure S8b. To measure its scattering parameters, two SMA connectors are used to connect its input and output ports with port 1 and port 2 of a vector network analyzer (VNA) (Keysight N9926A). From the results given in Figure S8c, we see that the reflection coefficients at the input port (S_11_) are below -10 dB from 5.1 to 6.2 GHz for the four coding states. In the frequency range between 5.4 and 5.6 GHz, the transmission magnitudes from port 1 to 2 (S_21_) are above -4.4 dB, and the variations between the four coding states are less than 0.4 dB. In addition, the 2-bit phase-shifting behavior is exhibited within the bandwidth by the transmission phase curves plotted in Figure S8d, from which the stable 90°-intervals can be observed. Because the phase shifter is a reciprocal device, the S_22_ and S_12_ curves are almost the same as the above results. As we check the measured reflection magnitudes of the prototype shown in Figure S8c, we find that the maximum value at 5.5 GHz is less than the result here by about 0.7 dB, which can be explained by the loss of the guiding and coupling paths in the unit cells. Additionally, the reflection magnitudes of the prototype drop dramatically as the frequency moves away from 5.55 GHz, which is attributed to the relatively narrow operating bandwidth of the radiators, which will be presented in the next section.


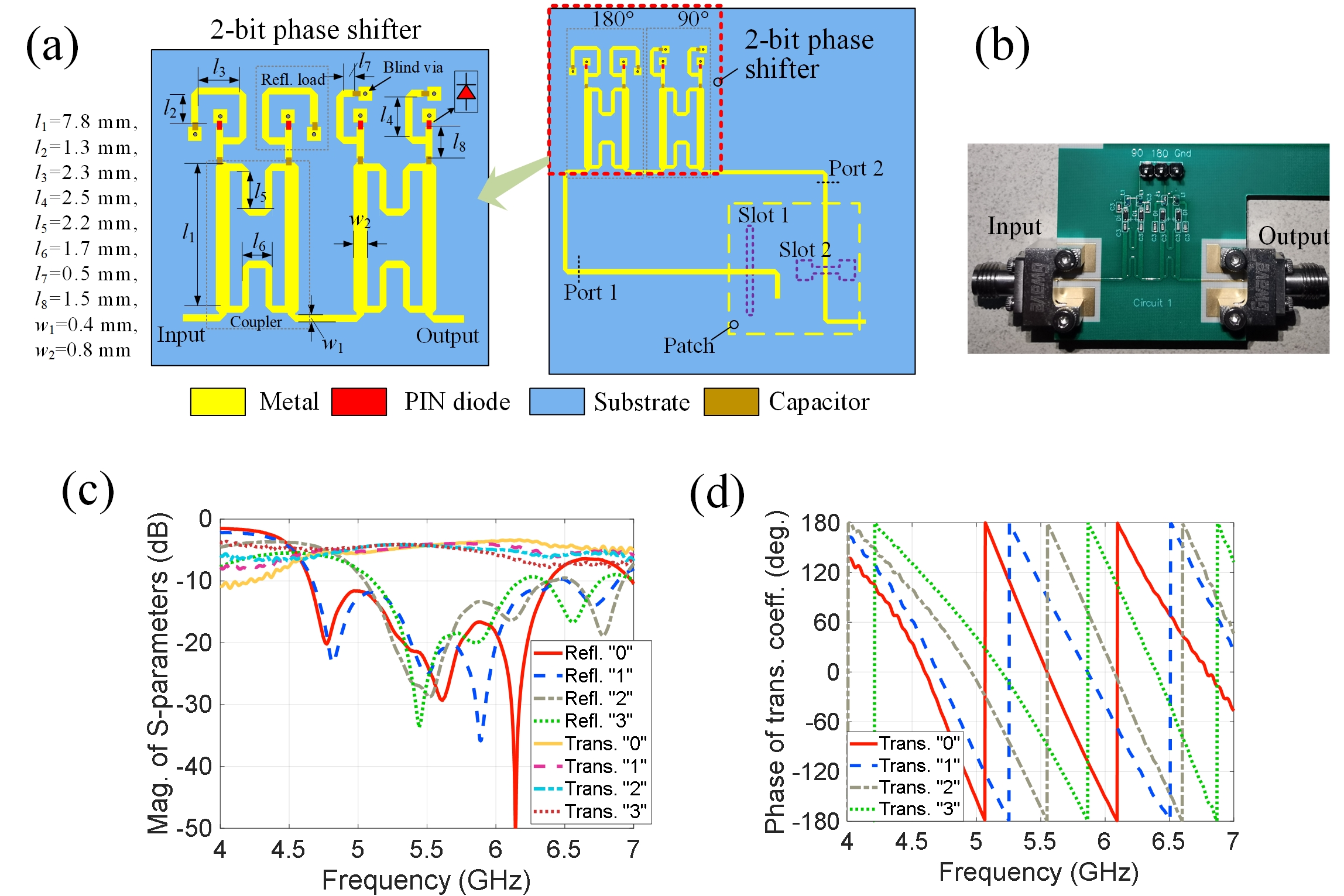


**Figure S8.** (a) Structure of the 2-bit phase shifter used in the metasurface unit cell, including two reflection-type phase shifters. (b) Picture of the 2-bit phase shifter. (c)-(d) Measured properties of the 2-bit phase shifter under the four coding states. (c) Reflection and transmission magnitudes under the four coding states. (d) Transmission phases under the four coding states.

## S12. Performances of the radiators of the metasurface unit cell

Radiating performances of a metasurface unit cell, without the phase shifter, are simulated using CST. Two wave ports are connected to the microstrips on the bottom layer, respectively, as labeled as ports 1 and 2 in Figure 8b in the main text. Figure S7a plots the reflection coefficients at the two ports and the coupling coefficients between them. It is observed that the bandwidth with S_11_ and S_22_ less than -10 dB is from 5.4 to 5.6 GHz. It is also found that the coupling coefficients are almost larger than 30 dB, showing a satisfactory isolation between the two polarizations. The scattering parameters of the two ports are measured using the VNA, and the results agree quite well with the simulations, as seen in Figure S9a. Figures S9b and c plot the simulated far-field radiation patterns of the patch when it is excited by the wave ports set at port 1 and port 2 in Figure 8b. We observe that the maximum directivities of about 6.2 dBi are achieved under the two polarizations. The main beams are slightly away from the broadside direction by about 6°, which can be explained by the square patch that is not at the center of the unit cell. This problem would be solved when a bunch of the unit cells are arranged into an array with a much larger ground.


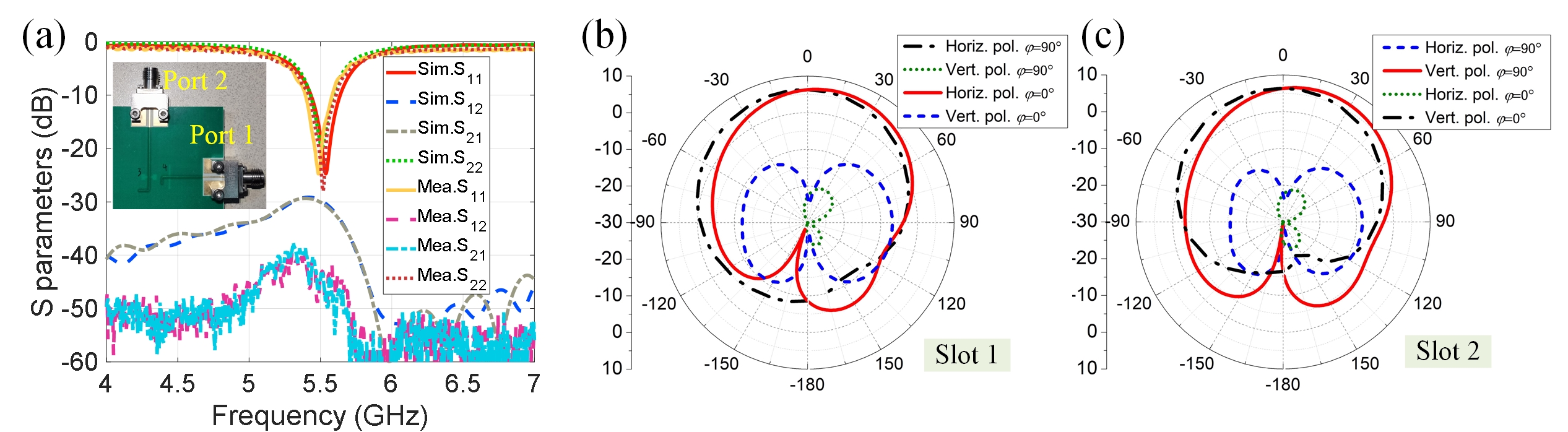


**Figure S9.** (a) Simulated and measured scattering parameters of the two ports of the radiators. (b)(c) Simulated far-field radiation patterns of the radiators excited by Slot 1 and 2, respectively.

## S13. Anomalous reflection performances of the metasurface

In this section, the far-field scattering performances of the metasurface with 5×10 unit cells are measured under the control of static coding sequences. Because of the temporally unchanged phase distributions on the panel in each case, the echo signals contain the incident fundamental frequency only. Here we assume that the reflection magnitudes of the cells are identical, and their reflection phases are , where *m* and *n* indicate their positions on the metasurface, so the far-field scattering patterns can be calculated using^[6]^

, (S6)

where is the field function of a unit cell, *D* is the period of each cell, $k=2\pi/\lambda$ is wave number in free space, and $\theta$ and are the elevation and azimuthal angles, respectively. For simplicity, the metasurface is divided into 10 columns along the *x*-axis, and each column with 5 unit cells is controlled by the same signal. When phase gradients are distributed on the panel, the incident wave is deflected to anomalous angles on the *xoz*-plane, which can be calculated by^[7]^

, (S7)

where $\lambda_{c}$ is the wavelength at 5.5 GHz, $n_{i}$ is the refraction index in free space, and is the phase gradient along the *x*-direction.

The measurement setup is the same as the nonlinear beam-steering measurement, which is shown in Figure S11b. The measured normalized far-field scattering patterns under the control of four coding sequences are presented in Figure S10. In Figure S10a with the homogeneous phase distribution, the incident wave is reflected normally. Due to the shading effect of the transmitting horn, the reflected signals are scattered again with a diverged beam. When the sequence is periodic “0123012301” and “0011223300”, respectively, theoretical deflection angles are 22.9° and 11.2°. We can see from Figure S10b and c that the scattering beams are deflected to 22° and 14°, respectively. As we set the sequence to “1111333311”, the incident wave is supposed to be split into two symmetric beams with ±11.2°, and the measured results in Figure S10d show the beam directions are 11.5° and -12.5°, respectively. The discrepancies between the theory and measurement can be attributed to the impact of the transmitting horn and the finite number of unit cells on the panel. Nevertheless, the 2-bit reflection phase shifting ability of the metasurface is experimentally demonstrated, which lays the groundwork for the time-domain controlling in the following work.

The scanning range of the harmonic beams generated by the metasurface can be estimated. The period of the unit cell is 0.64$\lambda_{c}$, which is the wavelength of the incident wave. Since the modulation frequency of the controlling signal is much smaller than the frequency of the incident wave, that is, $f_{0}\ll f_{c}$, the harmonic frequencies of interest are close to $f_{c}$, that is, $f_{h}=f_{c}\pm kf_{0}\approx f_{c}$. According to the general Snell’s law, the deflection angle of the scattering waves, which results from the phase gradient on the metasurface, can be obtained by

. (S8)

Here $\lambda_{h}$ is the harmonic wavelength; $dx=0.64\lambda_{h}$ is the period of the unit cell; is the phase difference between the unit cells, whose range is [–π, π]. Therefore, the harmonic beam-scanning range is

$\frac{\lambda_{h}}{0.64\lambda_{h}}\frac{-\pi}{2\pi}\leq\sin(\theta_{h})\leq\frac{\lambda_{h}}{0.64\lambda_{h}}\frac{\pi}{2\pi}$ , (S9)

which is $-51.4^{\circ}\leq\theta\leq51.4^{\circ}$.


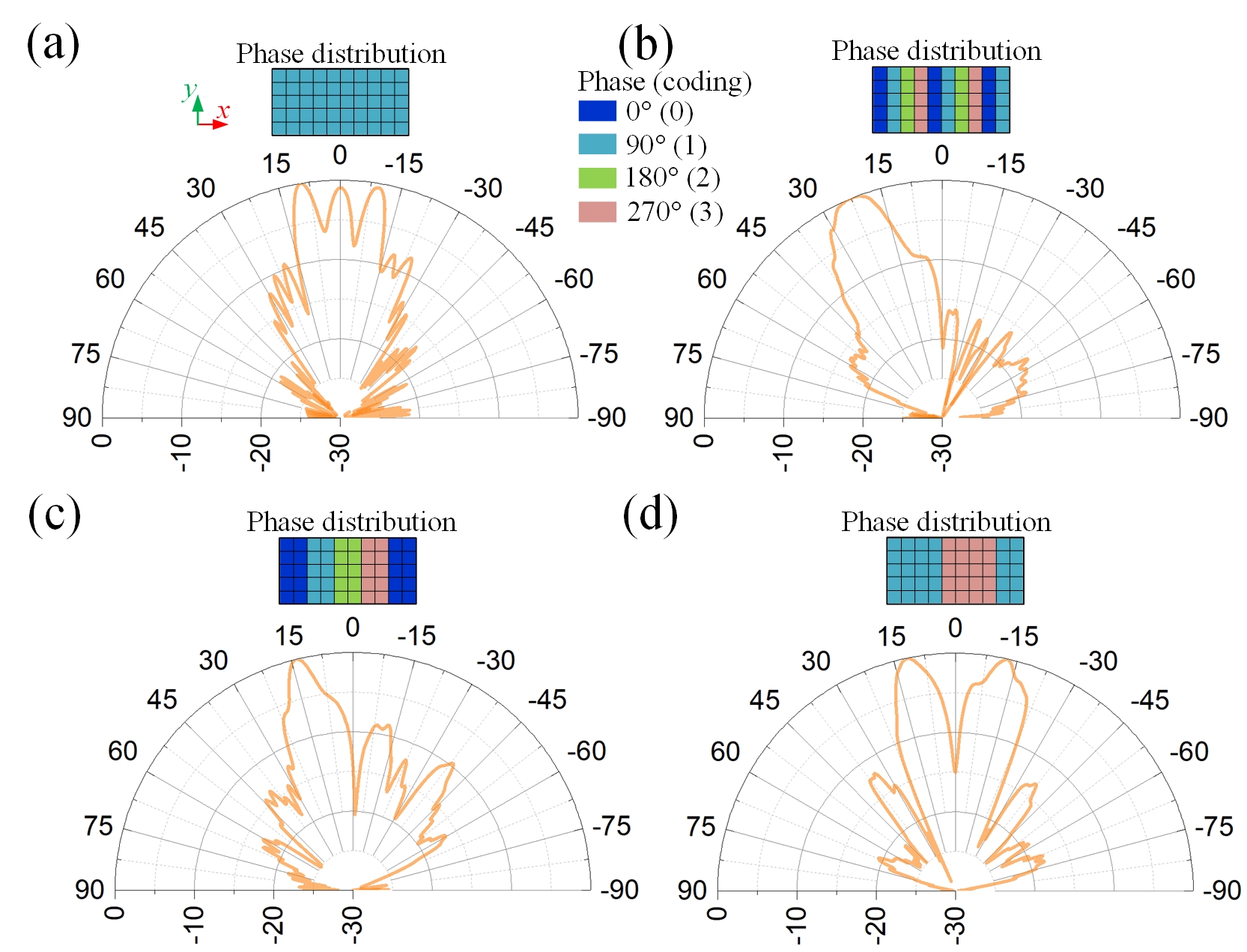


**Figure S10.** Measured far-field scattering patterns of the metasurface when it is controlled by statistic coding sequences. (a) The coding states of all the unit cells are identical. (b) The coding sequence is “0123012301”. (c) The coding sequence is “0011223300”. (d) The coding sequence is “1111333311”.

## S14. Experimental setups

**Measurement setup for the reflection coefficient of the 2-bit metasurface.**

The reflection properties of the array are measured in a microwave anechoic chamber. The measurement setup is shown in Figure S11a. All the unit cells behave identically under the control of the digital module. The metasurface, which is embedded in a large windowed absorbing screen, is placed in front of two linearly polarized horn antennas by 5 meters. The two horns are placed under the orthogonal polarizations, connected to port 1 and port 2 of a vector network analyzer (VNA) (Agilent N5230C), respectively. The reflection coefficients of the metasurface are obtained by reading S_21_ from the VNA. A metallic plate with the same size as the metasurface is also measured under the same condition, and its reflection coefficients are used to calibrate the results of the prototype.

**Measurement setup for the harmonic beam steering.**

The measurement setup is illustrated in Figure S11b. A signal generator (Agilent E8257D) is employed to provide a signal at 5.5 GHz with a power level of 20 dBm, which is radiated by an *x*-polarized transmitting horn antenna. The horn and the metasurface are mounted on a supporting board, and their distance is 1.3 meters. The radiation direction of the horn is perpendicular to the metasurface. The board is rotated with a mechanical turntable from -90° to 90° at increments of 0.5°. Another horn antenna under the *y*-polarization is located 2 meters away from the center of the metasurface and functions to receive the scattered signals and feed them to a signal analyzer (Agilent N9010A). The received signals at the harmonic frequencies are recorded, and the far-field scattering patterns on the *xoz*-plane can be plotted by rotating the board. The photograph of the measurement is presented in Figure S11d.

**Measurement setup for the beam deflection and wireless communication via various harmonics.**

The measurement setup is illustrated in Figure S11c. Unlike the setup in Figure S11b, the postures of the transmitting *x*-polarized horn and the metasurface are fixed; two *y*-polarized horns are employed as receiving antennas. Horn A is connected to the SDR kit, and Horn B is connected to the signal analyzer. Baseband signal processes, including signal synchronization, demodulation, and decoding, are accomplished using a computer, which is connected to the kit through a universal serial bus (USB) cable. Note that the positions of the two receiving antennas are not necessarily fixed. Because the beam directions of the harmonics can be changed dynamically, the receiving antennas should be placed at the correct positions to get satisfactory performances. The distance between the receiving antennas and the metasurface is 2 meters. The photograph of the measurement environment is presented in Figure S11e.


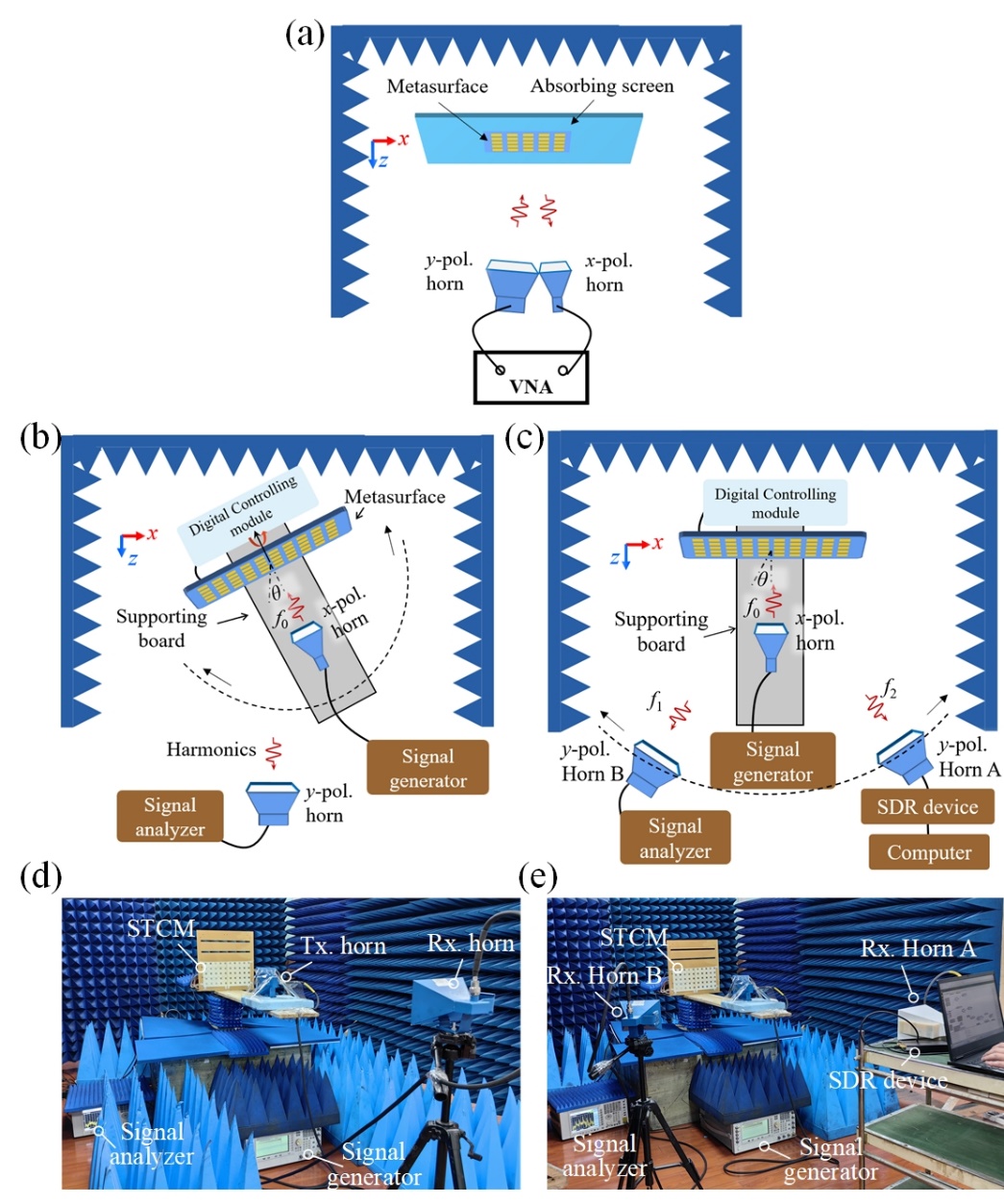


**Figure S11. (**a)-(c) Configuration of the nonlinear measurement setup. (a) Measurement for the reflection coefficient of the 2-bit metasurface. (b) Measurement for the harmonic beam steering. (c) Measurement for the beam deflection and wireless communication via various harmonics. (d) Photograph of the measurement environment for harmonic beam steering. (e) Photograph of the measurement environment for the joint beam deflection and wireless communication via various harmonics.

References

1. J. Y. Dai, J. Zhao, Q. Cheng, and T. J. Cui, *Light Sci. Appl.* **2018**, *7*, 90.
2. J. Y. Dai, J. Yang, W. Tang, M. Z. Chen, J. C. Ke, Q. Cheng, S. Jin, and T. J. Cui, *Appl. Phys. Rev.* **2020**, *7*, 041408.
3. S. R. Wang, J. Y. Dai, Q. Y. Zhou, J. C. Ke, Q. Cheng, and T. J. Cui, *Nat. Commun.* **2023**, *14*, 5377.
4. L. Zhang, X. Q. Chen, S. Liu, Q. Zhang, J. Zhao, J. Y. Dai, G. D. Bai, X. Wan, Q. Cheng, G. Castaldi, V. Galdi, and T. J. Cui, *Nat. Commun.* **2018**, *9*, 4334.
5. G. Castaldi, L. Zhang, M. Moccia, A. Y. Hathaway, W. X. Tang, T. J. Cui, and V. Galdi, *Adv. Funct. Mater.* **2021**, *31*, 2007620.
6. T. J. Cui, M. Q. Qi, X. Wan, J. Zhao, Q. Cheng, *Light Sci. Appl.* **2014**, *3*, e218.
7. N. F. Yu, P. Genevet, M. A. Kats, F. Aieta, J. P. Tetienne, F. Capasso, Z. Gaburro, *Science* **2011**, *334*, 333.
